# DeepSpecN: A new hybrid method combining PROSPECT-PRO and Conv-Transformer to estimate leaf nitrogen content from leaf reflectance

**DOI:** 10.1016/j.plaphe.2025.100125

**Published:** 2025-12-18

**Authors:** Shuai Yang, Anirudh Belwalkar, Dong Li, Yufeng Ge, Tao Cheng, Fei Wu, Longkang Peng, Daoliang Li, Kang Yu

**Affiliations:** a Precision Agriculture Lab, School of Life Sciences, Technical University of Munich, Freising, 85354, Germany; b National Innovation Center for Digital Fishery, China Agricultural University, China; c Key Laboratory of Smart Farming Technologies for Aquatic Animal and Livestock, Ministry of Agriculture and Rural Affairs, China Agricultural University, Beijing, 100083, China; d Beijing Engineering and Technology Research Center for Internet of Things in Agriculture, Beijing, 100083, China; e College of Information and Electrical Engineering, China Agricultural University, Beijing, 100083, China; f Department of Biological Systems Engineering, University of Nebraska-Lincoln, Lincoln, NE, 68583, United States; g Center for Plant Science Innovation, University of Nebraska-Lincoln, Lincoln, NE, 68588, United States; h National Engineering and Technology Center for Information Agriculture (NETCIA), Nanjing Agricultural University, Nanjing, China; i Department of Land Surveying and Geo-Informatics, The Hong Kong Polytechnic University, Hong Kong, China

**Keywords:** Leaf nitrogen estimation, Domain shift in machine learning, Radiative transfer model, Transformer architecture, Hybrid physical–AI modelling, Hyperspectral reflectance

## Abstract

Accurate, non-destructive quantification of leaf nitrogen content (LNC) is crucial for monitoring crop health and growth. Traditional empirical methods require extensive in-situ data for training, while physically-based methods are limited by ill-posed inversion, and hybrid methods suffer from domain shift between in-situ and simulated data. To overcome these limitations, this study introduces DeepSpecN, a novel hybrid method for maize LNC estimation using leaf-scale hyperspectral bidirectional reflectance. Without requiring in-situ data for training, DeepSpecN combines four key components: continuous wavelet transform (CWT) for reducing specular reflection, PROSPECT-PRO for simulating training data, an improved Transformer model for feature learning, and a spectral similarity-based sample selection method for selecting more valuable training samples. DeepSpecN and other methods, including physically-based methods, non-parametric regression based hybrid methods, and parametric regression methods based on vegetation indices (VIs), were validated using bidirectional reflectance data from 1724 maize leaves. The results showed that, when trained on representative samples, DeepSpecN achieved the highest estimation accuracy among all the methods (RMSE ​= ​0.247 ​g/m^2^, R^2^ ​= ​0.665). The sample selection strategy mitigated the effects of domain shift by identifying representative training samples with high spectral similarity from the simulated database. Furthermore, the results showed that the Chlorophyll (Chl)-based empirical formulas estimated maize LNC more accurately than those based on leaf protein content. Moreover, the validation results on four different crop species confirm the generalizability of DeepSpecN. Our findings demonstrate the potential of hybrid methods that utilize bidirectional reflectance spectra, developed by addressing the domain shift issue, to improve the LNC estimation accuracy.

## 1 Introduction

Nitrogen is one of the essential macronutrients for plants and is an important component of proteins, nucleic acids, and chlorophyll. For this reason, leaf nitrogen content is directly linked to the plant's photosynthetic efficiency, cell growth and division capabilities, as well as its overall growth and development [1]. The LNC can vary due to environmental heterogeneity. This variation is driven by biological factors such as plant species, phenology, and leaf developmental stage, as well as by abiotic factors like nutrient availability and water [2]. Traditional methods for measuring LNC involve laboratory chemical analysis, which are both expensive and time-consuming. By contrast, hyperspectral measurement offers an economical, efficient, and non-destructive analytical method, which has become a standard practice in precision agriculture for its ability to assess crop conditions without causing damage [3]. Estimating leaf N using hyperspectral reflectance is feasible because it leverages the relationship between N and spectral absorption features. N-sensitive bands can be identified by examining the bending and stretching of chemical bonds within leaves at particular wavelengths [4].

LNC estimation approaches using hyperspectral reflectance can be grouped into three broad categories: empirical, physical and hybrid methods [5]. Empirical methods are based on the statistical relationships between leaf biological traits and leaf reflectance, subdividing into parametric regressions and non-parametric regressions [6]. The parametric regression method includes simple regression with spectral VI from the determined spectral region [7]. The non-parametric regression methods mainly include machine learning (ML) models [[8], [9], [10]]. In the LNC estimation, the majority of existing VIs are formulated based on spectral bands from the visible and near-infrared (VNIR) region. This approach is predominantly driven by the established N-Chl relationships and the pronounced absorption features of Chl within the visible spectrum [11]. Although empirical methods are widely used, they lack robustness and transferability. This limitation arises because the calibration of these empirical relationships is typically based on a limited dataset, which may not adequately represent the variability and complexity across different environments or conditions [12]. Physically-based methods are typically based on radiative transfer models (RTMs), which simulate the optical properties such as spectral reflectance of leaves which simulate the optical properties such as spectral reflectance of leaves as a function of leaf biophysical and chemical properties. The inference of model variables is based on established knowledge encapsulated in RTMs and spectral variables [13]. Among all RTMs, PROSPECT is the most widely used RTM for simulating the leaf's directional-hemispherical reflectance and transmittance spectra across the 400–2500 ​nm wavelength range, based on the surface-unit content of various chemical constituents with specific absorption coefficients [14]. Running the PROSPECT model in inverse mode allows the determination of leaf traits by minimizing a cost function (or merit function), which involves comparing measured and simulated leaf reflectance [15]. Unlike empirical methods, the performance of RTMs in retrieving leaf constituents is less dependent on crop species and environments. Therefore, it has greater advantages in robustness and transferability [16]. However, the ability of PROSPECT to predict LNC is often limited due to the ill-posed nature of the inversions as different leaf trait combinations may yield similar leaf reflectance [17].

Hybrid methods integrate empirical and physical approaches, utilizing the strengths of both to address limitations and enhance estimation performance [6]. Physical models can generate large datasets containing simulated leaf traits and associated spectral reflectance. Statistical models efficiently extract relationships from spectral data and can manage large datasets effectively. These simulated datasets can be used to train empirical models by imposing physical constraints and providing plausible explanations, enabling them to learn complex patterns between simulated spectra and underlying leaf traits, thereby expanding their applicability [18]. Féret et al. [19] developed PROSPECT-PRO, a PROSPECT variant model that estimates leaf protein content by portioning total leaf dry matter into protein and carbon-based constituents (CBC). PROSPECT-PRO treats protein and CBC as retrievable parameters, and it has been calibrated under the principle that proteins and CBC together account for the total leaf mass. Protein is the primary nitrogen-containing biochemical property, with its absorption features mainly in the shortwave infrared (SWIR) region. The PROSPECT-PRO allows the SWIR to fully realize its potential in estimating LNC, opening up a new avenue for indirectly estimating LNC. In recent years, hybrid methods have demonstrated substantial potential for application in crop N modeling. Several studies utilizing hybrid methods have sought to estimate LNC, either indirectly or directly, by integrating leaf biochemical and biophysical traits retrieved from the PROSPECT and PROSPECT-PRO models with empirical models, resulting in high accuracy [20,21]. Although these studies yielded promising results, these models that rely on in-situ data may be considerably constrained when measured data is limited or unavailable as prior information [22,23]. Recent efforts have focused on developing hybrid models that are trained solely on simulated data to overcome this limitation. Chakraborty et al. [24] proposed a hybrid model by combining PROSPECT-PRO and Gaussian process regression, which was trained solely on simulated data and then validated on measured data. It showed promise and the viability of simulation data-based training for trait estimation in data-scarce environments, with reasonable accuracy in predicting almond LNC (RMSE ​= ​0.03 ​mg/cm^2^, R^2^ ​= ​0.54), underscoring the viability of simulation-based training for accurate trait estimation in data-scarce environments. Such work also highlights the necessity of developing alternative modeling models trained exclusively on simulated data to enable precise LNC estimation, thereby reducing reliance on the availability of in-situ data to enhance the generalizability of these models.

At the leaf-scale, specular reflection is a major obstacle preventing the transfer of knowledge learned from simulated data to in-situ data in hybrid models [25]. The PROSPECT model requires directional hemispherical reflectance factor (DHRF) or transmittance factor (DHTF) spectra as input, measured using an integrating sphere through a complex process. Therefore, DHRF and DHTF spectra are mainly used in theoretical studies and are impractical for large-scale applications. In contrast, bidirectional reflectance factor (BRF) spectra obtained via a leaf contact probe are more efficient, cost-effective, and widely applicable [25,26]. In the integrating sphere measurements, light undergoes multiple reflections before reaching the sensor, while leaf contact probes directly observe the leaf. The irregular surface of leaves produces specular reflection when the incident and viewing angles are aligned [27]. The primary distinction between DHRF and BRF arises from specular reflection on the leaf surface [28], leading to uncertainties when applying the PROSPECT model to BRF spectra.

The difference in data distribution between simulated and measured spectra may introduce additional uncertainty to the assessment of leaf biophysical and biochemical traits [29,30]. In the domain adaptation field of ML, when the training data does not accurately reflect the distribution of the validation data, the performance of the trained model may decline when applied to the test data. This phenomenon is known as “domain shift”. It is also why empirical models typically perform well on specific samples but have limited applicability for retrieving leaf traits in different plant species or under different growth conditions [31,32]. There have been only a few studies that addressed the domain shift issue for estimating biophysical and biochemical plant traits and for N estimation using canopy-level hyperspectral reflectance [[33], [34], [35], [36]]. In addition to the specular reflection mentioned above, another possible cause of the domain shift is the simulation of unrealistic samples. While a large sample size helps to generalize the ML model, errors may occur in the PROSPECT simulated data because the RTM input parameters are sampled independently, potentially leading to the simulation of unrealistic samples [22]. These samples may interfere with, rather than contribute to, the training of non-parametric regression models. Nevertheless, the domain shift issue is still underexplored in the context of LNC estimation; for instance, it is unknown whether training a model only within the simulated data domain can be directly applicable to the in-situ leaf reflectance measurement data domain when transferred to other species. Moreover, a large simulated dataset increases the time and computational burden required for model training, whereas a small simulated dataset may fail to encompass a diverse range of leaf trait combinations. Thus, how to select representative training samples from a large simulated dataset is still unclear.

The majority of LNC estimation studies currently utilize traditional ML models [37]. Considering that hyperspectral data has a large number of consecutive bands, adjacent bands often exhibit high correlation. This can lead to extremely high input feature dimensions, containing a significant amount of redundancy or noise, and ML models may lack sufficient capacity to address these issues [6], making it difficult to fully capture the rich information hidden within hyperspectral data. Moreover, these models rely heavily on manual feature engineering [38]. In contrast, deep learning (DL) models can automatically extract complex features. Multi-layer neural networks are capable of modeling arbitrarily complex nonlinear functions, making them more suitable for handling high-dimensional data [6]. Some DL models, such as long short-term memory (LSTM) [39] and Transformer [40], can process hyperspectral reflectance and model global dependencies between spectral bands. Previous studies based on empirical methods [41,42] have shown promising results in LNC estimation using DL models. Yu et al. [41] achieved high accuracy (RMSEP ​= ​0.307 ​%, R^2^ ​= ​0.903) in predicting leaf nitrogen concentration in oilseed rape using stacked auto-encoders and a fully connected neural network. Chen et al. [42] yielded accurate predictions (NRMSE ​= ​9.06 ​%, R^2^ ​= ​0.790) for apple tree LNC by combining a deep neural network with phenological information. While these findings indicate a significant potential for DL models in LNC estimation, further investigation is needed to explore advanced architectures for LNC prediction across plant species using hyperspectral data.

To address the aforementioned challenges, we propose a new hybrid method, DeepSpecN, which integrates CWT for reducing specular reflection, the PROSPECT-PRO model, a Transformer architecture for feature learning, and a spectral similarity-based sample selection method for identifying more valuable simulated training samples, aiming to estimate LNC in four crops: maize, wheat, rice, and sorghum. The specific sub-objectives are outlined as follows:

(1) Compare the proposed DeepSpecN with (a) five variations of the PROSPECT-PRO model; (b) hybrid methods based on five different non-parametric regression models; and (c) parametric regression models based on thirty different VIs.
(2) Evaluate the LNC estimation accuracy through empirical formulas that utilize Chl content and leaf protein content.
(3) Assess how varying cost functions and the choice of representative samples from the source domain, determined by spectral similarity, influence model performance.
(4) Test the generalizability of DeepSpecN across different crops and conduct ablation experiments to analyze the contributions of the sample selection strategy and the improved Transformer model to LNC prediction.

## 2 Materials and methods

### 2.1 Data description

#### 2.1.1 Main dataset in maize

The maize (Zea mays) leaf samples for this study were collected from the University of Nebraska-Lincoln between 2018 and 2020. Measurements were conducted across various developmental stages, ranging from late vegetative growth to flowering. For plants that had reached the flowering stage, samples were collected from the second, third, and fourth leaves below the flag leaf. In contrast, for those in the late-vegetative phase, three leaves immediately beneath the most recently emerged leaf were selected for analysis. More information about the dataset can be found in Refs. [43,44].

**Fig. 1:**
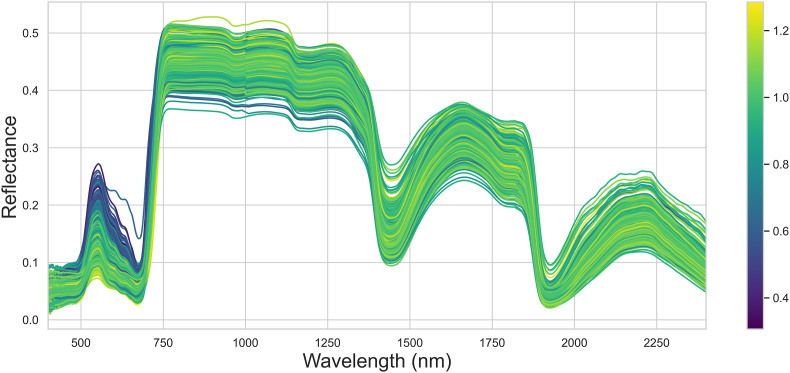
Reflectance spectra of 1724 maize leaf samples.

Hyperspectral reflectance measurements were conducted for 1724 maize leaves using a benchtop spectroradiometer (FieldSpec4, Malvern Panalytical Ltd.) equipped with a contact probe, positioned at leaf level. The device covers a spectral range from 350 to 2500 ​nm, with spectral resolutions of 3 ​nm for the 350–1000 ​nm band and 10 ​nm for the 1000–2500 ​nm band. Raw data were resampled to 1-nm intervals. To account for within-leaf variability, three separate spectral readings were taken for each leaf: one at the tip, one at the middle, and one at the base, ensuring that the midrib area was avoided. Finally, the average of the nine measurements was taken as the final spectral reading. Every 15 ​min, a white panel with a reflectance of 99 ​% (Spectralon, Labsphere Inc.) was used to recalibrate the instrument during data collection. To prevent backscattered light from passing through the leaf, a dark panel with an average reflectance of 2 ​% was placed beneath the scanning area. Fig. 1 presents the reflectance spectra of 1724 maize samples, displaying characteristic patterns typical of healthy vegetation. The spectral curve reveals moderate reflectance in the visible range (approximately 400–650 ​nm), high reflectance in the near-infrared region (around 700–1000 ​nm), and distinct water absorption bands near 1340–1450 ​nm and 1790–2030 ​nm. These features confirm that the selected maize leaves are healthy and suitable for the intended analysis. In addition, we noted that LNC has a significant influence on the reflectance changes in the spectral region of 400–700 ​nm, which is also sensitive to Chl content.

#### 2.1.2 Leaf sample processing and LNC determination

After spectral sampling, the maize leaf samples were immediately processed and measured. Leaf area (LA, ${\text{cm}}^{2}$) was measured using a leaf area meter (LI-3100, LI-COR Biosciences). After measuring the fresh weight (FW, g) of the leaves using a digital balance, they were placed in an oven at 50 ​°C and dried to a constant weight over 72 ​h, after which the dry weight (DW, g) was recorded. The mass-based leaf N concentration (N%) was measured using the Dumas method with a LECO FP428 N analyzer. Based on Eq. (1), Eq. (2), and Eq. (3), we further calculated the area-based LNC ($g/m^{2}$), leaf equivalent water thickness ($C_{w}$, $g/{\text{cm}}^{2}$), and leaf mass per area ($C_{m}$, $\mu g/{\text{cm}}^{2}$).

(1) LNC=(0.01×N%×DW)/(LA×0.0001)

(2) $$C_{w}=\left(FW-\text{DW}\right)/\text{LA}$$

(3) $$C_{m}=\text{DW}/\text{LA}$$

In this study, the LNC estimation model training was based on the PROSPECT-PRO simulated dataset. This approach necessitates that both the simulated and in-situ datasets share the same target variable. However, since LNC is not an input parameter in PROSPECT-PRO, this limitation precludes the direct estimation of LNC from the simulated dataset. To address this issue, leaf Chl content and protein content serve as the two important proxies representing the N status of crops [45]. Chl content is highly correlated with spectral bands located in the VNIR (400–800 ​nm), while certain spectral bands in the SWIR (1000–2500 ​nm) show sensitivity to leaf protein content [22]. Thus, we calculated LNC by two different approaches using leaf protein content ($C_{prot}$, $g/{\text{cm}}^{2}$), leaf Chl content ($C_{ab}$, $\mu g/cm^{2}$), and leaf dry matter content ($C_{m}$, $g/{\text{cm}}^{2}$), which are obtainable from PROSPECT-PRO. The calculation formulas are shown in Eq. (4) and Eq. (5) [46,47].

(4) $$\mathrm{LN}\;C_{a}=0.0231\times C_{ab}+0.0071\times C_{m}$$

(5) $$\mathrm{LN}\;C_{b}=C_{prot}/4.43$$

where Eq. (4) is referred to as the nitrogen allocation model, while Eq. (5) is termed the protein-to-nitrogen conversion model [45]. In this study, considering the edge effect of the CWT and the response range of leaf biochemical parameters to leaf spectra, we used VNIR and SWIR to retrieve $\mathrm{LN}\;C_{a}$ (450–800 ​nm and 1000–2450 ​nm), and only SWIR was used to retrieve $\mathrm{LN}\;C_{b}$ (1000–2450 ​nm). Other spectral ranges were discarded due to their insensitivity to LNC or low signal quality.

#### 2.1.3 Validation dataset in wheat, rice and sorghum

To validate the applicability of the newly proposed DeepSpecN modelling framework across different crops, we used datasets in wheat, rice [48], and sorghum [44], evaluated our method. The descriptive statistics of leaf traits of these crops are presented in Table S8.

### 2.2 Workflow of the LNC estimation methods

This study compared three modeling methods, including the physical-model LUT inversion, VI-based parametric regression, and the hybrid model for LNC estimation. The workflow consists of two stages: Stage 1 involves the utilization of PROSPECT-PRO to generate a large simulated dataset. Next, the three methods were applied to estimate the LNC of in-situ data, using the large simulated dataset for model calibration. Stage 2 employs the spectral similarity-based sample selection method to extract representative samples from the large simulated dataset. The parameters in the VI-based parametric and non-parametric regression based hybrid methods were calibrated to estimate the LNC of in-situ data, utilizing the selected representative samples.

### 2.3 Physically-based LUT inversion methods

We used the look-up table (LUT)-based inversion approach to retrieve maize LNC, which aims to minimize the discrepancies between measured and RTM-simulated leaf reflectance using a cost function and, in turn, determines the leaf traits from the LUT. Here, we ran five variants of the PROSPECT model for LUT-based inversion, including: (a) PROSPECT-PRO [19]; and (b) PROSPECT-PRO followed by the subtraction of leaf surface specular reflectance (PROREF) for LUT-based inversion [49,50]; (c) PROSPECT-PRO extended to account for additional spectral variability induced by directional effects and variation in leaf orientation (PROCOSINE) [51]; (d) the coupling of PROSPECT-PRO with CWT (PROCWT) [50]; and (e) PROSPECT-PRO coupled with spectral derivatives and similarity metrics (PROSDM) [52]. All five methods took the measured leaf BRF spectra as input to produce a singular optimal solution, while they differ in their cost functions.

For PROREF, we opted for reflectance at 1925 ​nm, rather than the standard implementation of 445 ​nm following Li et al. [50]. For PROCOSINE, the light incident angle $\theta_{i}$ and the illumination zenith angles $\theta_{s}$ were set at 12° [53]. We used iterative numerical optimization to retrieve the specular parameter corresponding to each in-situ spectrum. Since the systematic amplitude of the leaf BRF spectrum is higher than that of the DHRF spectrum [25], subtracting the corresponding specular parameter from each in-situ spectrum, the in-situ spectrum based on pseudo-DHRF. For PROCWT, the first derivative of the Gaussian function was chosen as the mother wavelet, with decomposition scales of 2^3^, 2^4^, and 2^5^ for the wavelet transformation, denoted as PROCWT-S3, PROCWT-S4, and PROCWT-S5. For PROSDM, four inversion methods were developed using first-order spectral derivative (FD) and second-order spectral derivative (SD), along with two spectral similarity metrics: Euclidean distance (ED) and Manhattan distance (MD). These methods were designated as PROSDM-FD-ED, PROSDM-SD-ED, PROSDM-FD-MD, and PROSDM-SD-MD, respectively.

### 2.4 Parametric regression methods based on hyperspectral VIs

VIs have been extensively utilized for the estimation of LNC [6]. Common types include the simple ratio (SR), normalized difference (ND), and their variants [25,54]. The formulas for these indices are as follows:

(6) $$SR=\frac{R_{\lambda 1}}{R_{\lambda 2}}$$

(7) $$ND=\left(R_{\lambda 1}-R_{\lambda 2}\right)/\left(R_{\lambda 1}+R_{\lambda 2}\right)$$

Where $R_{\lambda 1}$ and $R_{\lambda 2}$ are the reflectance values of a spectrum at wavelength $\lambda_{1}$ and $\lambda_{2}$, respectively. For the SR indices, Sims et al. [49] proposed the modified SR, which subtracts the reflectance value at the 445 ​nm wavelength from both the numerator and the denominator. We used 1925 ​nm instead of 445 ​nm based on our tests that indicated the LNC estimation accuracy of mSR based on 1925 ​nm is higher than that of mSR based on 445 ​nm (refer to Table S5). The expression for the mSR index is as follows:

(8) $$mSR=\frac{R_{\lambda 1}-R_{1925}}{R_{\lambda 2}-R_{1925}}$$

To explore which VIs can resist the effects of specular reflection, we selected a variety of hyperspectral VIs from previous studies, including SR, ND, mSR, and other types of indices. Table S12 lists the VIs used in this study. We combined each VIs individually with linear regression as the main regression factor, and coefficient fitting was conducted on the simulated data. Finally, the linear regression model fitted on the simulated data was applied to the in-situ data.

### 2.5 DeepSpecN model

**Fig. 2:**
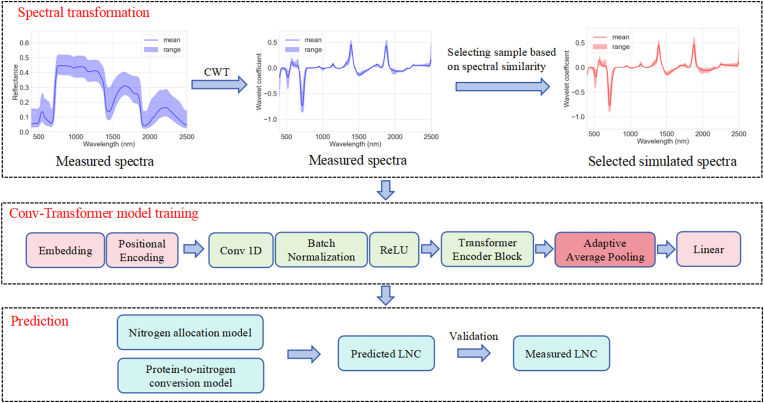
The architectural diagram of DeepSpecN.

We propose a new method named DeepSpecN. It involved three phases (Fig. 2). In the first phase, a spectral similarity-based sample selection method is employed to select training samples from the simulated spectra (source domain) that are most relevant to the in-situ spectra (target domain). The second phase aims to minimize the discrepancies between DHRF-based simulated data and BRF-based in-situ data using CWT. Finally, these selected simulated samples are used to train the Conv-Transformer model we proposed, with final validation conducted on the in-situ data.

#### 2.5.1 Spectral transformation

Modeling results can vary based on the spectral data transformation methods employed. Therefore, in addition to CWT, we also tested three other spectral transformation methods. We examined four distinct spectral transformation methods to reduce the influence of specular reflection: (1) CWT based on first-order Gaussian function as the wavelet basis function, and scales as 2^3^, 2^4^, and 2^5^, applied to simulated and measured spectrum, expressed as $P_{CWT-S3}$, $P_{CWT-S4}$, and $P_{CWT-S5}$; (2) Using PROCOSINE on the measured spectrum, and generating a pseudo-DHRF spectrum, expressed as $P_{PROCOSINE}$; (3) FD applied to simulated and measured spectrum, expressed as $P_{FD}$; (4) Based on PROREF, subtracting the reflectance at 1925 ​nm from both the simulated spectrum and measured spectrum, expressed as $P_{REF}$. Furthermore, we utilized a control group ($P_{0}$) that did not undergo any transformation to evaluate the performance of the four spectral transformation methods. We assumed that the difference between leaf clip-based BRF measurements and PROSPECT-simulated DHRF measurements is consistent across wavelengths, in accordance with Walter-Shea et al. [55].

#### 2.5.2 Proposed Conv-Transformer model

Considering the characteristics of hyperspectral reflectance, we employed a novel DL model, which integrates one-dimensional convolutional neural network (1D-CNN) for local feature extraction, and Transformer for long-range dependency capture [56]. Since this network architecture is modified based on Transformer, we name it Conv-Transformer. The overall architecture of the proposed model is depicted in Fig. 2.

**Fig. 3:**
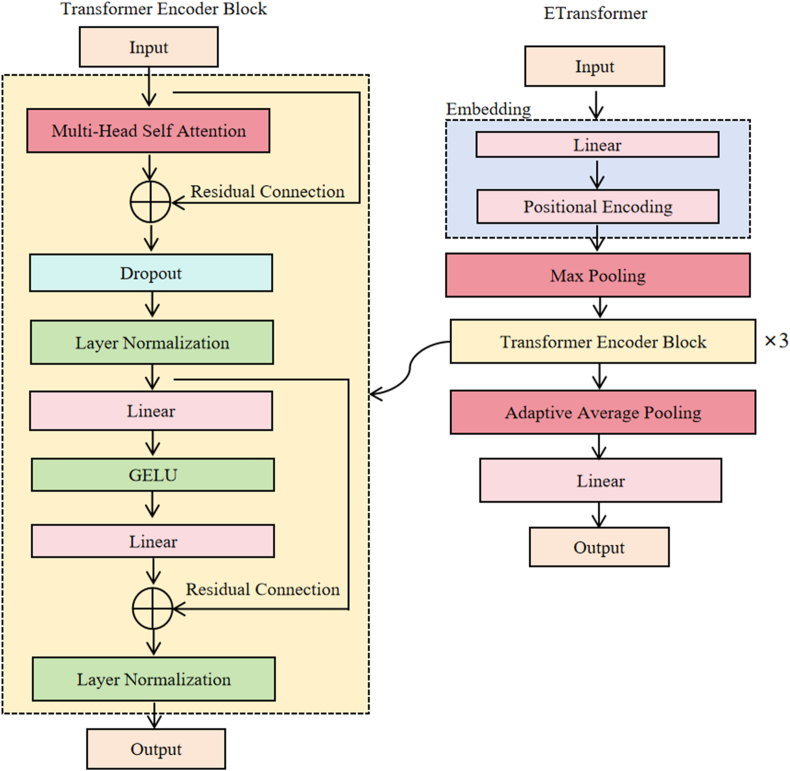
The network architecture of the ETransformer.

Conv-Transformer is composed of the following key components: one embedding layer, one 1D-CNN layer, and three Transformer encoder blocks. The embedding layer projects hyperspectral reflectance into a higher-dimensional space, enabling the network to learn richer representations. Additionally, the positional encoding layer attaches position information to each band in the sequence, allowing the subsequent modules to capture dependencies at different scales between “adjacent bands” and “distant bands”. The 1D-CNN layer progressively extracts local features from the hyperspectral data by capturing the correlations between adjacent spectral bands. These convolutional operations slide over the spectral band sequence, helping to alleviate the high inter-band redundancy inherent in hyperspectral signals. Utilizing strided convolutions for down-sampling effectively reduces the sequence length by half, which subsequently lowers the computational cost of the following Transformer self-attention. Furthermore, this approach allows convolutional kernels to optimize the merging of neighboring frames to enhance information retention. After the 1D-CNN layer, the ReLU activation function is applied to introduce non-linearity, which enables the model to represent and learn complex, non-linear patterns present in hyperspectral data [57]. Additionally, dropout is employed to improve the robustness of the model by reducing overfitting. Finally, the three Transformer encoder blocks focus on capturing the global dependencies and latent patterns within the high-dimensional hyperspectral data, further enhancing the model's capacity to represent long-range relationships. Each Transformer encoder block consists of a multi-head self-attention layer followed by a feed-forward neural network. Residual connections are applied around the attention module and feed-forward neural network, improving gradient flow, alleviating gradient explosion, and improving the training stability of the model. By stacking three Transformer encoder blocks, the model gains sufficient depth and iterative processing capability, enabling it to capture both simple and highly complex global patterns in high-dimensional hyperspectral data, thereby enhancing its overall learning capacity. Additionally, to investigate the impact of convolution on our model's performance, we designed an ablation experiment: we removed the 1D-CNN layer from the Conv-Transformer and substituted it with max pooling layer to maintain the same downsampling capability as the 1D-CNN layer. We refer to this modified model as ETransformer (Fig. 3).

Attention mechanism is a widely used technique in DL models, particularly in fields such as natural language processing, computer vision, and speech recognition [58]. Its core idea is to enable the model to dynamically focus on different parts of the input data [40]. It calculates the similarity between all elements directly and sums according to weight (Eq. (9)).

(9) $$Attention\left(Q,K,V\right)=soft\;\max\left(\frac{QK^{T}}{\sqrt{d_{k}}}\right)V$$

**Fig. 4:**
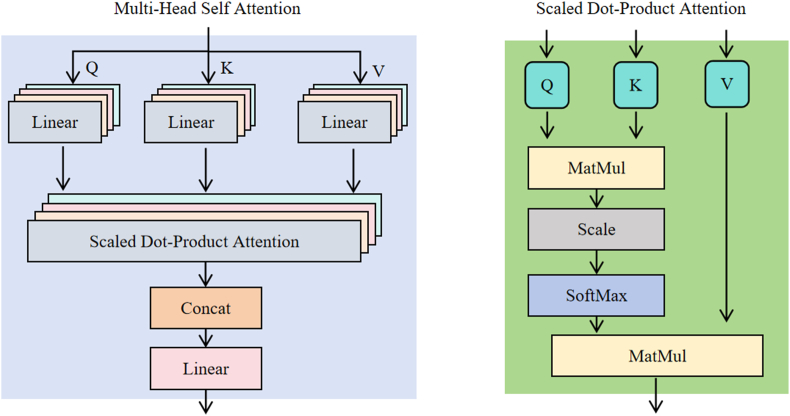
The architecture of the attention layer.

Where Q, K, V represent the query matrix, key matrix, and value matrix, respectively, $d_{k}$ denoting the dimensions for a single head. The core idea of multi-head attention is to extend the attention mechanism into multiple heads, where each head can independently learn different parts of the input information and process them in parallel [59]. The architecture of multi-head attention is shown in Fig. 4. First, Q, K, and V are mapped into multiple subspaces through different linear transformations. Since the Q, K, and V of each head are independently linearly transformed, multiple heads can capture different relationships within the input. Finally, the results of the multiple heads are concatenated and mapped to the output dimension via a fully connected layer. Therefore, multi-head attention is able to learn more features and relationships than a single attention mechanism. By computing the attention of multiple heads in parallel, the model can understand the input sequence from multiple perspectives, thereby enhancing its expressive power, especially when processing long sequence data. In this study, the number of attention heads in multi-head attention was set to 4, and the hidden layer dimension was set to 128. In addition to Conv-Transformer and ETransformer, we also tested another DL model and three ML models that have performed well in other domains for comparison: LSTM, Light gradient boosting machine regressor (LGBM), Support vector regression (SVR), Least absolute shrinkage and selection operator (Lasso). We used a 3-layer LSTM model (hidden layer dimension ​= ​128), with the dimension of the hidden layer set to 128 [60]. The optimal combination of hyperparameters for the ML models was determined using the grid search method (detailed parameter settings can be found in Table S6).

#### 2.5.3 Spectral similarity-based sample selection method

Domain adaptation is a set of techniques aimed at enabling a model trained on a source domain to perform well on a different target domain [61]. In this study, our goal is to train the model in the source domain of simulated data and then apply it to the target domain of in-situ data. First, spectral transformation methods were employed to reduce the distributional differences between simulated and in-situ data. Then, a sample selection method based on spectral similarity, corresponding to the spectral transformation approach, was used to filter potentially beneficial simulated samples from a large simulated database for model training. Specifically, we used different cost functions to calculate spectral similarity. The cost functions correspond to the following physical models: standard PROSPECT-PRO inversion (Eq. (10)), PROREF (Eq. (11)), PROCOSINE (Eq. (12)), PROCWT-S3 (Eq. (13)), PROCWT-S4 (Eq. (14)), PROCWT-S5 (Eq. (15)), and PROSDM-FD-ED (Eq. (16)).

(10) $$\min_{P_{0}}=\sqrt{\sum_{\lambda =1}^{\lambda =n}{\left(BRF_{\lambda }^{m}-DHRF_{\lambda }^{s}\right)}^{2}}$$

(11) $$\min_{P_{REF}}=\sqrt{\sum_{\lambda =1}^{\lambda =n}{\left[\left(BRF_{\lambda }^{m}-BRF_{1925}^{m}\right)-\left(DHRF_{\lambda }^{s}-DHRF_{1925}^{s}\right)\right]}^{2}}$$

(12) $$\min_{P_{PROCOSINE}}=\sqrt{\sum_{\lambda =1}^{\lambda =n}{\left[BRF_{\lambda }^{m}-\frac{\cos\left(\theta_{i}\right)}{\cos\left(\theta_{S}\right)}\left(DHRF_{\lambda }^{s}+f_{\lambda }\right)\right]}^{2}}$$

(13) $$\min_{P_{CWT-S3}}=\sqrt{\sum_{\lambda =1}^{\lambda =n}{\left(W{\left(BRF_{\lambda }^{m}\right)}_{2^{3}}-W{\left(DHRF_{\lambda }^{S}\right)}_{2^{3}}\right)}^{2}}$$

(14) $$\min_{P_{CWT-S4}}=\sqrt{\sum_{\lambda =1}^{\lambda =n}{\left(W{\left(BRF_{\lambda }^{m}\right)}_{2^{4}}-W{\left(DHRF_{\lambda }^{S}\right)}_{2^{4}}\right)}^{2}}$$

(15) $$\min_{P_{CWT-S5}}=\sqrt{\sum_{\lambda =1}^{\lambda =n}{\left(W{\left(BRF_{\lambda }^{m}\right)}_{2^{5}}-W{\left(DHRF_{\lambda }^{S}\right)}_{2^{5}}\right)}^{2}}$$

(16) $$\min_{P_{FD}}=\sqrt{\sum_{\lambda =1}^{\lambda =n}{\left(BRF_{\lambda 1}^{m}-DHRF_{\lambda 1}^{s}\right)}^{2}}$$

Where $BRF_{\lambda }^{m}$ is the measured BRF spectra, $DHRF_{\lambda }^{s}$ is the simulated DHRF spectra, measured at wavelength λ. $W{\left(BRF_{\lambda }^{m}\right)}_{a}$ and $W{\left(DHRF_{\lambda }^{s}\right)}_{a}$ are the wavelet coefficients of the measured BRF and simulated DHRF spectra at scale a when the wavelet basis function is the first derivative of the Gaussian function, respectively. n is the number of wavebands.

**Fig. 5:**
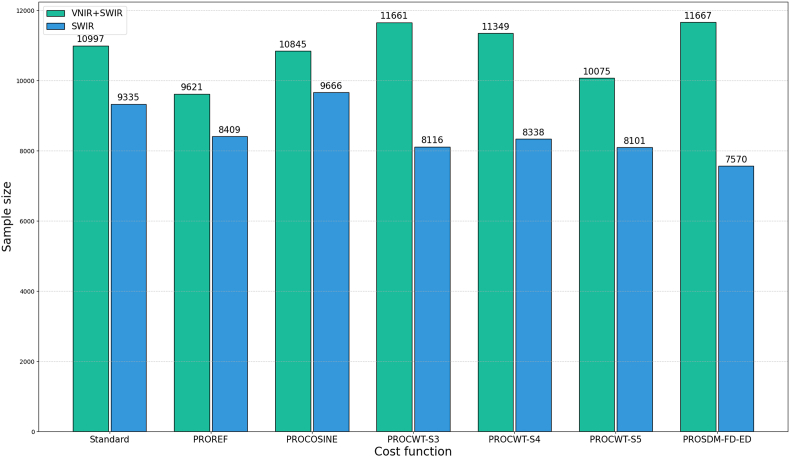
The number of samples in different T100 datasets.

For each in-situ spectrum, the top 100 simulated spectra with the highest spectral similarity values were selected from the simulated large database containing 200,000 simulations. These selected spectra were merged into a new training database (with duplicate samples removed), referred to as T100 dataset (representative simulated dataset). Spectral transformation was applied to the T100 dataset and the in-situ data according to the respective cost functions. Fig. 5 shows the number of samples in T100 constructed using different cost functions. We used T100-i (i corresponds to the physical model's name for different cost functions) to distinguish between different T100 datasets. The process of filtering training data using cost functions effectively performs a targeted optimization of the training dataset, aligning it more closely with the distribution and characteristics of the in-situ data. This approach enhances the model's generalization capability on in-situ data. Essentially, it is a domain adaptation technique that effectively bridges the distribution gap between simulated and in-situ data.

### 2.6 Parameter setting in the PROSPECT-PRO simulations and training

We used PROSPECT-PRO to generate 200,000 combinations of all input parameters based on uniform distribution, creating a simulated database (LUT) that corresponds to hyperspectral reflectance in the 400–2500 ​nm range for a specific parameter space. The parameter ranges for PROSPECT-PRO and PROCOSINE were set based on the input ranges of the in-situ dataset (Table 1). Table S7 presents the statistical information of leaf biochemical parameters from the simulated and in-situ datasets, ensuring that the range of leaf parameters in the simulated data fully encompasses that of the in-situ data. For PROCOSINE, the iterative numerical optimization to retrieve the specular parameter corresponding to each in-situ spectrum was implemented using MATLAB's ‘lsqcurvefit’ function, the trust-region reflective algorithm was applied to minimize the squared error between the measured reflectance and the reflectance simulated by the PROSPECT-PRO model. The inversion performance of the model was evaluated by Root Mean Squared Error (RMSE), Mean Absolute Percentage Error (MAPE), and Coefficient of Determination (R^2^ Score).

**Table 1:** The ranges of input parameters for PROSPECT-PRO and PROCOSINE. Initial value, $C_{m}$, $\theta_{i}$, and Bspec were only used for PROCOSINE. Prot and CBC were only used in PROSPECT-PRO.

Parameters	Unit	Minimum	Maximum	Initial value
Leaf structure parameter ($N_{struct}$)	unitless	1	3	2
Leaf Chl content ($C_{ab}$)	$\mu g/cm^{2}$	10	80	40
Leaf carotenoid content ($C_{xc}$)	$\mu g/cm^{2}$	1	20	10
Leaf anthocyanin content ($C_{anth}$)	$\mu g/cm^{2}$	0	0	0
Leaf water content ($C_{w}$)	$g/cm^{2}$	0.005	0.04	0.02
Leaf protein content ($C_{prot}$)	$g/cm^{2}$	0.0001	0.002	–
Carbon-based constituents (CBC)	$g/cm^{2}$	0.0005	0.01	–
Leaf dry matter content ($C_{m}$)	$g/cm^{2}$	0.0006	0.012	0.006
Light incident angle ($\theta_{i}$)	°	12	12	12
Specular parameter (Bspec)	unitless	−0.2	0.6	0.2

The stochastic gradient descent algorithm and the Adam optimiser [62] were utilized for training the DL model. The training batch size for the DL model employing the entire simulated dataset of 200,000 simulations was determined to be 256, while for the T100 dataset, it was set at 64. The epochs were set at 1000, with an initial learning rate of 0.001. A simulated dataset comprising 1724 simulations, each closely matching a corresponding measured in-situ spectrum, was derived from the original 200,000 simulations. The sampling methodology employed the LUT linked to the cost function for each spectral transformation method. This dataset was additionally employed as a validation set to apply early stopping criteria, which ceases training when there is no improvement in model performance, and for grid search to identify optimal parameter combinations for ML models.

Our computations were conducted on a system with dual AMD EPYC 9534 64-Core Processors (2.45 ​GHz, 128 total cores), 384 ​GB RAM, and a single NVIDIA A40 GPU running Windows Server 2022 Standard Edition with CUDA 12.4 version installed. We used Python 3.9.21 and PyTorch 2.5.0 as our DL framework. The ML model was implemented in Scikit-learn library 1.6.1. In this study, the simulated data generated by PROSPECT-PRO and the numerical optimization process based on PROCOSINE were realized in MATLAB 2023b (The Mathworks, Inc., Natick, MA, USA). All other computations were performed using Python 3.9.21.

## 3 Results

### 3.1 Illustration of spectra after spectral transformation

**Fig. 6:**
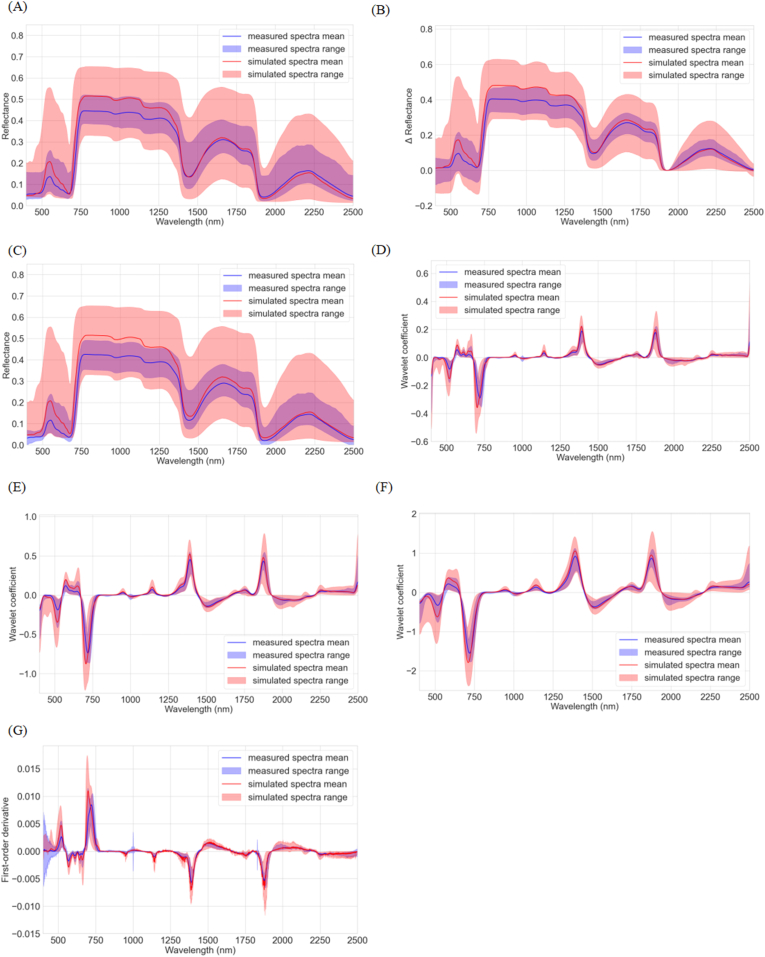
The mean, maximum and minimum reflectance of the measured spectra and simulated spectra after applying different spectral transformation methods: (A) $P_{0}$, (B) $P_{REF}$, (C) $P_{PROCOSINE}$, (D) $P_{CWT-S3}$, (E) $P_{CWT-S4}$, (F) $P_{CWT-S5}$, and (G) $P_{FD}$.

Fig. 6 illustrates the range and mean of the simulated spectra as well as those of the in-situ spectra after spectral transformation. From the original reflectance (Fig. 6A), it can be observed that the amplitude of the simulated DHRF reflectance spectra is slightly higher than that of the measured BRF spectra. Within the 450–2450 ​nm range, the difference between the mean lines of the simulated DHRF and measured BRF reflectance spectra accounts for approximately 13.35 ​% of the measured BRF reflectance spectra. This difference is approximately 38.19 ​% in the 500–700 ​nm range, 14.57 ​% in the 750–1300 ​nm range, and 7.72 ​% in the 1300–2450 ​nm range. Thus, this discrepancy is more pronounced in the VNIR. As shown in Fig. 6D–G, CWT and FD reduce this discrepancy, bringing the mean lines of the transformed simulated and measured spectra closer together. Furthermore, some absorption features are enhanced. Considering that direct comparison of the amplitudes between measured BRF and simulated DHRF spectra is difficult, we hypothesize that enhancing absorption features could potentially mitigate the effects of domain shift and improve LNC estimation. The total range of the simulated spectra encompasses nearly the entire range of the in-situ spectra, highlighting the importance of a comprehensive representation of all potential scenarios for developing an effective training dataset.

### 3.2 Performance of parametric regression methods based on hyperspectral VIs

**Fig. 7:**
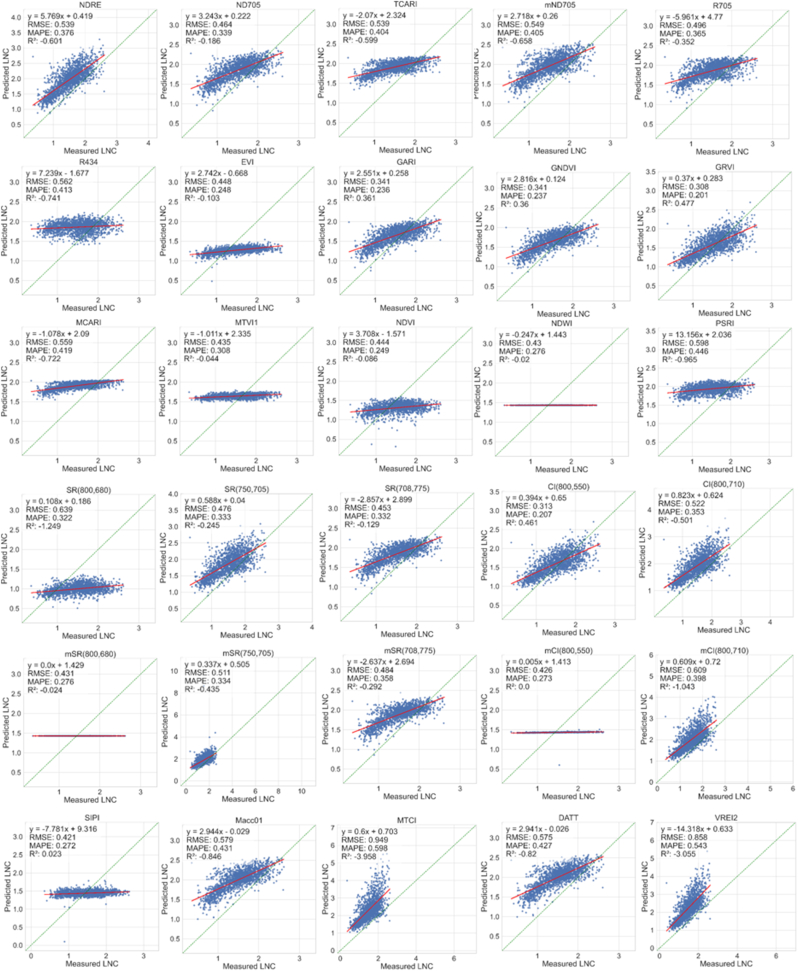
LNC estimation accuracy of the parametric regression method fitted on the entire simulated dataset using the nitrogen allocation model.

Fig. 7 shows the prediction results of the linear regression models comparing the performance of thirty different VIs using the entire simulated dataset. The results employing the nitrogen allocation model demonstrated that the linear regression models based on GARI, GNDVI, GRVI, and CI_800,550_ achieved higher accuracy than some physically-based methods, with R^2^ values of 0.361, 0.360, 0.477 and 0.461, respectively. Furthermore, all VIs exhibited insensitivity to LNC calculated using protein-to-nitrogen conversion model, with MAPE exceeding 75 ​% (see Table S3 for details).

**Fig. 8:**
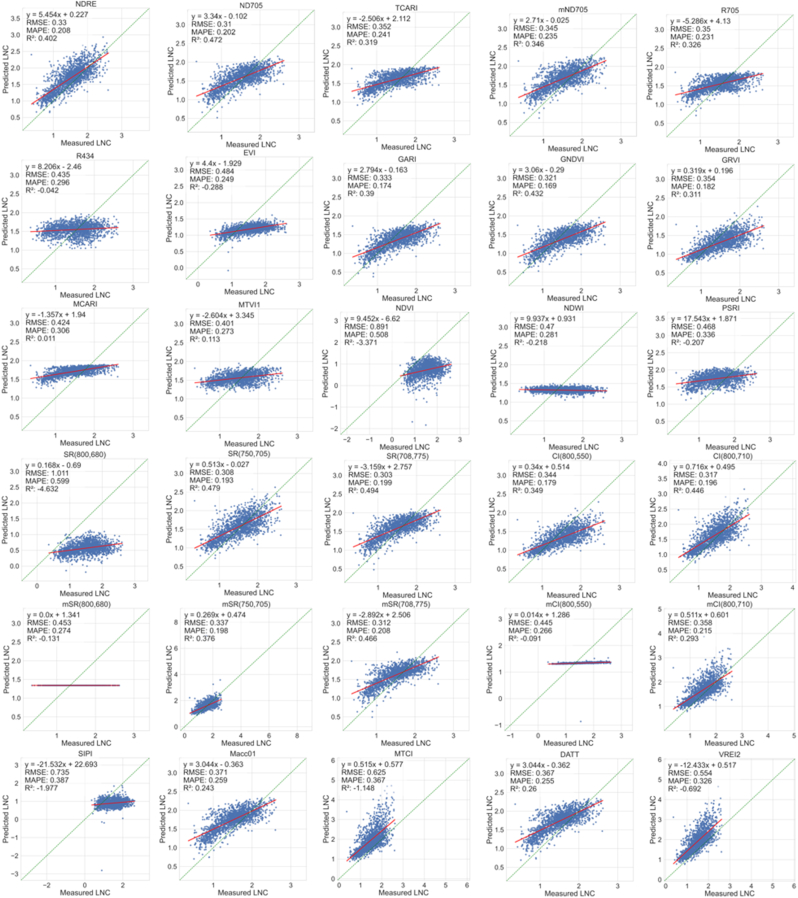
LNC estimation accuracy of the parametric regression method fitted on T100-Standard using the nitrogen allocation model.

Fig. 8 shows the performance of the linear regression models after parameter fitting on the T100 dataset. The results employing the nitrogen allocation model indicated that NDRE, ND705, GNDVI, SR_750,705_, SR_708,775_, CI_800,710_, mSR_708,775_, and mSR_708,775,1725_ achieved higher LNC estimation accuracy compared to some physical inversion methods, with all R^2^ values exceeding 0.4. Among these, SR_708,775_ achieved the highest accuracy (RMSE ​= ​0.303 ​g/m^2^, R^2^ ​= ​0.494). Additionally, most VIs showed improved estimation performance for LNC when compared to using the entire simulated dataset with 200,000 simulations. This suggests that, for parametric regression methods, the quality and representativeness of the samples are more important than the sample size.

### 3.3 Performance of non-parametric regression-based hybrid methods

**Fig. 9:**
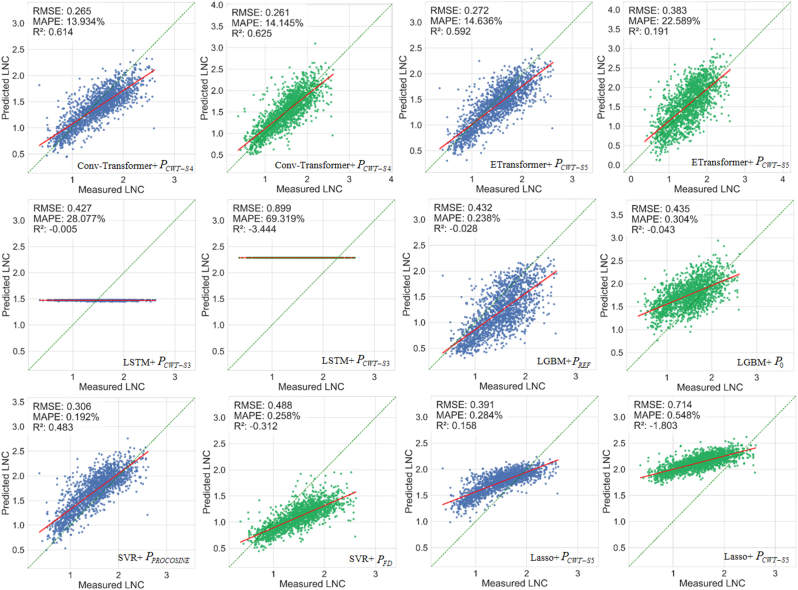
The optimal combination of each model and spectral transformation method to predict LNC using the entire simulated dataset for model training. Blue points represent the LNC of simulated spectra based on the nitrogen allocation model, while green points represent the LNC of simulated spectra based on the protein-to-nitrogen conversion model. The green dashed line represents the 1:1 line, and the red solid line represents the fitted line. The same notation is used in all the following figures unless stated otherwise.

Fig. 9 illustrates the optimal combination of each DL or ML model with the spectral transformation method using the entire simulated dataset with 200,000 simulations for model training. For the retrieval method based on the nitrogen allocation model, DL models generally outperformed ML models. For the nitrogen allocation model and protein-to-nitrogen conversion model, Conv-Transformer surpassed all other models in LNC estimation accuracy (RMSE ​= ​0.265 ​g/m^2^, R^2^ ​= ​0.614; RMSE ​= ​0.261 ​g/m^2^, R^2^ ​= ​0.625). Hybrid models utilizing the nitrogen allocation model generally demonstrated better performance compared to those using the protein-to-nitrogen conversion model. We also compared the LNC estimation accuracy of five physically-based methods (Fig. S1), the PROCWT-S5 model achieved the best performance (RMSE ​= ​0.268 ​g/m^2^, R^2^ ​= ​0.603). PROCWT-S4 and PROSDM-FD-MD also achieved relatively high accuracy (RMSE ​= ​0.290 ​g/m^2^, R^2^ ​= ​0.536; RMSE ​= ​0.298 ​g/m^2^, R^2^ ​= ​0.512). Overall, the performance of hybrid methods is slightly higher than that of physically-based methods.

**Fig. 10:**
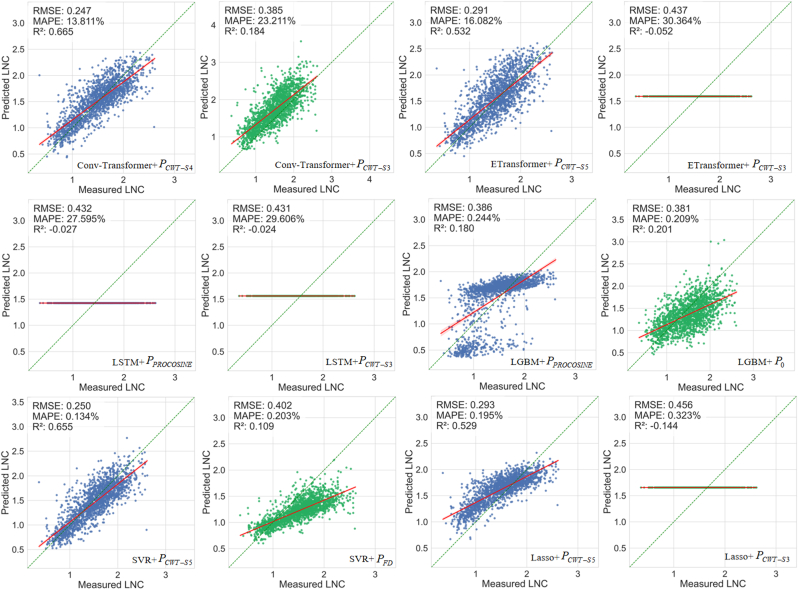
The optimal combination of each model and spectral transformation method to predict LNC using the T100 dataset for model training.

Fig. 10 demonstrates the optimal combination of each DL or ML model with the spectral transformation method using the T100 dataset for model training. The Conv-Transformer outperformed all other models in LNC estimation accuracy based on the nitrogen allocation model and the T100-PROCWT-S4 (RMSE ​= ​0.247 ​g/m^2^, R^2^ ​= ​0.665). Its LNC prediction performance, trained on only about 10,000 simulated samples, surpassed that achieved when training was conducted on the full simulated dataset. The LSTM shows consistently poor LNC estimation accuracy, regardless of whether it is trained on the entire simulated dataset or the T100 dataset. The LNC estimation accuracy of ETransformer, when trained on the entire simulated dataset and T100 dataset independently, exhibited minimal variation in its optimal combination. For the ML models, LGBM trained on the T100 dataset showed an improvement in LNC prediction performance compared to using the entire simulated dataset for training. When LGBM was combined with the T100-PROCOSINE dataset and the nitrogen allocation model (RMSE ​= ​0.386 ​g/m^2^, R^2^ ​= ​0.180), the R^2^ improved by 0.208. In contrast, when LGBM was combined with the T100-Standard dataset and the protein-to-nitrogen conversion model (RMSE ​= ​0.381 ​g/m^2^, R^2^ ​= ​0.201), the R^2^ improved by 0.244. The highest LNC estimation accuracy for SVR was achieved on the T100-PROCWT-S5 dataset and the nitrogen allocation model, yielding an RMSE of 0.250 ​g/m^2^ and an R^2^ of 0.655, which reflects an R^2^ improvement of 0.172 compared to training on the entire simulated dataset. Lasso achieves its best LNC estimation performance on the T100-PROCWT-S5 dataset and nitrogen allocation model (RMSE ​= ​0.293 ​g/m^2^, R^2^ ​= ​0.529), showing a substantial improvement over training on the entire simulated dataset (RMSE ​= ​0.391 ​g/m^2^, R^2^ ​= ​0.158). However, the sample selection strategy failed to improve the LNC prediction accuracy based on the protein-to-nitrogen conversion model.

### 3.4 The validation of DeepSpecN in different crops

**Fig. 11:**
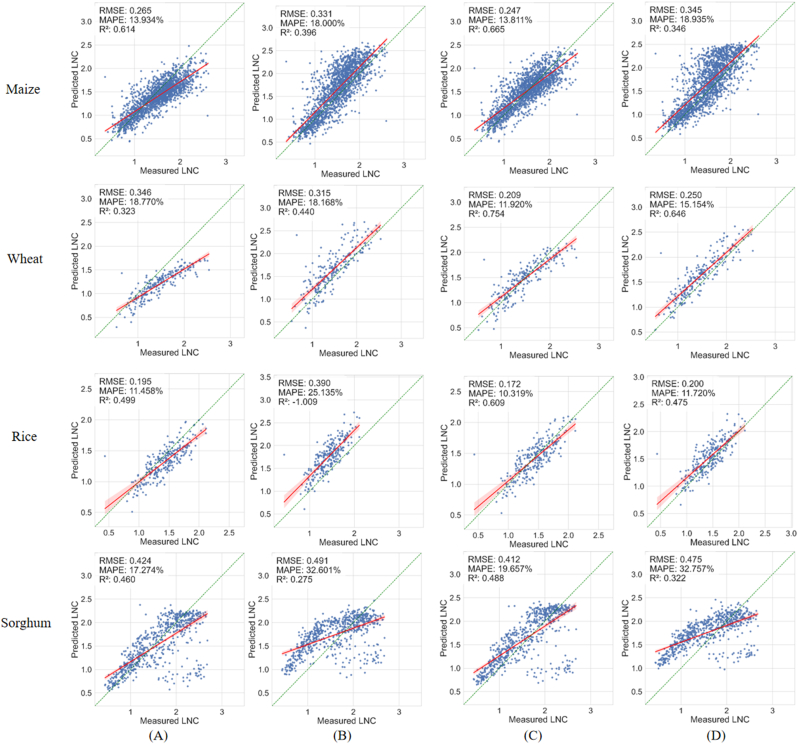
LNC prediction performance of DeepSpecN in four crops based on four model-ablation experiments. (A) Conv-Transformer trained on the entire simulated dataset, (B) ETransformer trained on the entire simulated dataset, (C) Conv-Transformer trained on the T100 dataset, (D) ETransformer trained on the T100 dataset.

To assess the contribution of the proposed spectral similarity-based sample selection method and the modifications to the Transformer model architecture on LNC prediction performance, we conducted an ablation study to separately evaluate their individual impacts on the LNC prediction performance (Fig. 11). We compared the models, before and after Transformer architecture modification, in different crops, as well as between the entire simulated dataset and the T100 dataset (Table S9 and Table S10). Notably, when trained on the T100 dataset, the Conv-Transformer further improves performance across all crops compared to the ETransformer: For maize, RMSE decreased from 0.345 to 0.247 and R^2^ increased from 0.346 to 0.665; For wheat, RMSE dropped from 0.250 to 0.209 and R^2^ rose from 0.646 to 0.754; For rice, RMSE decreased from 0.200 to 0.172 and R^2^ improved from 0.475 to 0.609; and for sorghum, RMSE reduced from 0.475 to 0.412 with R^2^ increasing from 0.322 to 0.488. Combining the sample selection strategy and the Conv-Transformer consistently yielded promising LNC prediction accuracy across four crops. These findings highlight the synergistic effect of the Conv-Transformer and the sample selection strategy, which together enhance the overall performance of the DeepSpecN framework.

## 4 Discussion

### 4.1 DeepSpecN for LNC estimation

Our study revealed that integrating the Conv-Transformer with the first derivative Gaussian wavelet at a decomposition scale of 2^4^, using the T100-PROCWT-S4 simulated dataset for training and validating on measured maize data, achieved LNC estimation accuracy that surpassed all other models (Table S2). CWT effectively captures the frequency patterns of hyperspectral reflectance across designated scale ranges, thereby enhancing the frequency signals associated with LNC [63]. In the processing of hyperspectral data using CWT, smaller-scale wavelet basis functions effectively capture high-frequency components of the signal, which generally represent local details. Larger-scale wavelet basis functions, in contrast, capture low-frequency components that represent global trends or long-term variations in the signal. In our study, maize hyperspectral data processed with CWT demonstrated more distinct local features at a decomposition scale of 2^4^. The convolutional kernels of the 1D-CNN effectively extracted local correlations by sliding along the frequency axis [64]. The improved local information allowed the 1D-CNN to extract higher-level features with greater efficacy. The integration of CWT, Transformer, and 1D-CNN effectively captured the intricate relationships between hyperspectral data and LNC. Moreover, the optimal wavelet scale for CWT may vary slightly depending on the crops. For example, as shown in Table S9, the optimal scale is 2^4^ for maize and wheat, 2^3^ for rice, and 2^5^ for sorghum. Our LNC estimation results with DeepSpecN are inferior to those from previous studies that employed hybrid methods. For example, Li et al. [22] integrated in-situ data and simulated data to train the model, achieving an R^2^ of up to 0.7 for the best estimation accuracy of maize LNC. Chen et al. [65] also combined in-situ data with simulated data for model training; when the in-situ data accounted for 90 ​%–100 ​% of the calibration dataset, the model achieved the best prediction accuracy for wheat LNC on the validation set, with R^2^ values ranging from 0.81 to 0.88. The reason for this is that, in these studies, in-situ data were partitioned into training and validation datasets to train DL or ML models, yielding high accuracy on the validation set. Nonetheless, the trained model was not tested on a dataset from a fully independent domain, as both the training and validation sets were derived from the same population.

We found that compared to the LUT approach, the combination of the spectral similarity-based sample selection method and hybrid methods demonstrated superior estimation accuracy. This is because physical models are typically based on ED calculations, where ED between points in high-dimensional space tends to become similar, leading to matching failures [66]. Moreover, ED encounters challenges in comprehensively considering complex interrelationships between different spectral bands [67]. Non-parametric regression based hybrid methods can construct multi-level feature representations, thereby automatically extracting high-dimensional features most relevant to LNC. These models are first trained on data generated by PROSPECT-PRO, enabling the DL or ML components to inherit certain constraints from physical models. This integration preserves the interpretability of physical models while leveraging the powerful feature learning of ML and DL, thereby enhancing overall inversion accuracy.

### 4.2 Impact of spectral similarity-based sample selection on LNC prediction

The selection of training samples based on spectral similarity is crucial for developing robust models and improving estimation accuracy [68]. Our results indicate that not all samples contribute positively to model prediction within a large simulated training dataset (Table S1 and Table S2). Similar findings have been reported in other fields, confirming that training samples contribute unequally to model performance. In the field of neural machine translation, Iter et al. [69] proposed using data selection techniques to identify suitable pre-training data from the source domain, guided by a limited set of target domain data. They showed that this approach, followed by additional training, can improve the model's generalization capabilities in the target domain. In tasks such as part-of-speech tagging, dependency parsing, and sentiment analysis, Liu et al. [70] showed that synchronously searching for training samples related to the target domain from the source domain during learning through reinforcement learning enables the task-specific model to learn better representations. Ma et al. [71] actively selected appropriate source samples to avoid negative transfer and significantly improved the accuracy of cross-domain keyword recognition. When the source domain contains many samples that have significant distributional differences from the target domain, enforcing feature alignment across the entire domain may lead to the model learning irrelevant features, thus reducing its generalization performance. Source domain samples that are more similar to the target domain can more accurately preserve the local feature structure, whereas global alignment might obscure such details.

Simulated data may cover an excessively large spectral parameter space, making it difficult for the model to learn features specific to in-situ data. Additionally, simulated data may include many out-of-distribution spectral features that are absent in in-situ data. The sample selection based on the spectral similarity method selects samples closer to the distribution of real-world measured data, reducing the distributional gap between simulated and in-situ datasets [52]. In essence, we do not actually use target domain data for training but primarily aim at improving learning efficiency – screening out simulations that are more reliable for the target task while avoiding learning from ill-posed simulations, enabling the DL model to concentrate more on what truly matters for real-world measured data and achieve a better performance [72]. As described by Danner et al. [73] and Chen et al. [74], the main limiting factor of the hybrid method is the discrepancy between the observed spectra and the simulated spectra. In leaf-scale studies, this discrepancy is primarily caused by specular reflection and the simplifications in the PROSPECT-PRO. Our study demonstrated that CWT and the spectral similarity-based sample selection method effectively reduced this discrepancy, leading to enhanced LNC estimation accuracy. Moreover, in the field of DL, overfitting is one of the main factors affecting model transferability [75]. In this study, we addressed this issue by using the LUT method to identify the simulated spectra most similar to the in-situ spectra for early stopping decisions. Training was terminated early when the model's performance on these simulated spectra no longer improved, thereby preserving the model's optimal state. This approach effectively avoids the problem of overfitting.

In this study, we selected the 100 simulated spectra most similar to each measured spectrum to construct the T100 dataset. Generally, the smaller the value of *x* in top-*x* is, the closer the selected simulations are to the target domain data distribution. However, the sample size that is too small might be insufficient to support stable training of DL models. On the other hand, when *x* is too large, the selected simulated samples deviate more from the target domain in distribution. Considering that DL models require a sufficient amount of data for training (though far less than the total simulated dataset), our experiments showed that setting *x* ​= ​100 strikes a good balance: it provides enough samples for stable model training while ensuring that the selected simulated data are more similar in distribution to the target domain than the full simulated dataset. Additionally, the number of samples in the T100 dataset varies depending on the number of measured samples. Therefore, it is necessary to adjust the batch size accordingly, i.e., reducing the batch size when fewer samples are selected and increasing it when more samples are available. As shown in Fig. 11, we also applied *x* ​= ​100 for datasets of different crops, and the results confirmed the rationale behind this choice.

### 4.3 Impact of LNC formulation on estimation accuracy

Our study revealed that the estimation accuracy of LNC based on the protein-to-nitrogen conversion model was relatively low (Table S9 and Table S10). There could be several potential factors contributing to this low estimation accuracy. One potential factor could be the weak absorption of proteins, which overlaps with the absorption characteristics of water and dry matter [48]. Another potential factor could be that the coefficient of the protein-to-nitrogen conversion model may not be suitable for some crops. Although the nitrogen allocation model achieved satisfactory performance in this study, the coefficients in the formula were calibrated using datasets from rice and wheat. For maize, recalibration on a maize dataset may be necessary. Additionally, the relationship between Chl and LNC is not always stable and can fluctuate significantly depending on the growth stage, environment, crop type, and the level of N fertilization applied [11]. These observations underscore the necessity for calibration tailored to specific crops and the enhancement of modelling strategies that consider the dynamic physiological and environmental factors affecting LNC estimation.

### 4.4 Limitations and future directions

While the proposed DeepSpecN modeling framework has been shown to be effective based on leaf-level hyperspectral data in four crops, future research is still needed to assess the robustness and transferability across crop species. Moreover, we anticipate further investigating the scalability of the proposed framework for canopy-level nitrogen modeling and multiple-trait estimation, and expanding its application to more crop types under diverse environmental conditions [[76], [77]].

The accuracy of DeepSpecN's LNC prediction also depends on the correct mapping between the simulated spectra and LNC. If this mapping fails to adequately capture the relationship between spectral reflectance and LNC for the target crop, the DL model will be unable to learn an effective representation from the simulated data. For this reason, the LUT inversion method based on the protein-to-nitrogen conversion model generally exhibits low LNC prediction accuracy across the four crops (Table S11). This results in a biased mapping between spectral reflectance and LNC among the samples selected by the spectral similarity-based sample selection method. In such cases, increasing the number of training samples may be more beneficial for model development (Table S10).

The widespread adoption of the hybrid modeling method is largely due to the open access and practicality of the PROSPECT model for simulating spectral characteristics across a wide range of plant species. However, if the calibration dataset for the PROSPECT model lacks representative samples from certain crop species, then its application to that species may result in systematic bias. For instance, as shown in Fig. 11, DeepSpecN exhibited relatively low predictive performance for sorghum LNC, potentially due to the insufficient sorghum representative data in the calibration dataset. Therefore, future research should focus on collecting more in-situ spectral measurements across diverse species and environmental conditions to refine model parameters and enhance its generalizability and accuracy in varied application scenarios.

## 5 Conclusions

This study proposed a novel hybrid method, DeepSpecN, which integrated CWT, the PROSPECT-PRO model, and an improved Transformer model to enhance the estimation of maize LNC. The results demonstrate that when trained on representative samples selected based on spectral similarity, DeepSpecN significantly outperforms alternative approaches. It surpasses five different physically-based methods, hybrid methods based on five different non-parametric regression methods, and parameter regression methods based on thirty VIs. Notably, DeepSpecN achieves an accuracy of RMSE ​= ​0.247 ​g/m^2^ and R^2^ ​= ​0.665 without requiring in-situ data for training. This emphasizes the importance of sample selection in addressing domain shifts. Furthermore, the results obtained on wheat, rice, and sorghum datasets confirmed the generalizability of DeepSpecN across different crop species. Overall, the study highlights the potential of hybrid methods that incorporate physical constraints, spectral similarity-based sample selection, and DL, for non-destructive crop N monitoring.

## General

We are grateful to the reviewers for providing valuable suggestions for this study.

## Author contributions

Shuai Yang: Conceptualization, Methodology, Writing - Original Draft. Anirudh Belwalkar: Writing - Review & Editing, Validation. Dong Li: Investigation, Writing - Review & Editing. Yufeng Ge: Data curation, Writing - Review & Editing. Tao Cheng: Data curation, Writing - Review & Editing. Fei Wu: Visualization, Formal analysis. Longkang Peng: Visualization, Formal analysis. Daoliang Li: Funding acquisition, Writing - Review & Editing. Kang Yu: Funding acquisition, Writing - Review & Editing, Supervision, Conceptualization, Resources.

## Funding

This work was supported by the ‘AmAIzed’ project funded by the AgroMissionHub, the 10.13039/501100001809National Natural Science Foundation of China (grant numbers 32373186), and the CAU-TUM joint PhD training program (2023-2025) funded by ​China Scholarship Council.

## Declaration of competing interest

The authors declare that they have no known competing financial interests or personal relationships that could have appeared to influence the work reported in this paper.

## Data Availability

Data collected and/or analyzed during this study can be obtained from the corresponding author upon reasonable request.
