# Supplementary material for: DeepSpecN: A new hybrid method combining PROSPECT-PRO and Conv-Transformer to estimate leaf nitrogen content from leaf reflectance
Source: Plant Phenomics. 2025 Dec 18;7(4):100125. doi: 10.1016/j.plaphe.2025.100125 (PMC13109343; doi:10.1016/j.plaphe.2025.100125)
Supplement: Multimedia component 1 [file mmc1.pdf]

Table S1: The LNC estimation accuracy of non-parametric regression method based on all simulated data.

Model	Evaluation indicator	Data preprocessing	RMSE	MAPE (%)	R^2
Conv-Transformer	LNC_a	P_0	0.311	17.151	0.467
		P_{REF}	0.282	15.579	0.561
		$P_{PROCOSINE}$	0.301	16.497	0.500
		P_{FD}	0.389	24.425	0.165
		P_{CWT-S3}	0.388	23.177	0.170
		P_{CWT-S4}	0.265	13.934	0.614
		P_{CWT-S5}	0.431	25.062	-0.022
	LNC_b	P_0	0.441	28.826	-0.071
		P_{REF}	0.442	28.359	-0.075
		$P_{PROCOSINE}$	0.453	29.394	-0.131
		P_{FD}	0.880	67.818	-3.265
		P_{CWT-S3}	0.372	23.103	0.236
		P_{CWT-S4}	0.261	14.145	0.625
		P_{CWT-S5}	0.338	19.073	0.369
ETransformer	LNC_a	P_0	0.365	18.470	0.266
		P_{REF}	0.323	16.560	0.426
		$P_{PROCOSINE}$	0.422	23.673	0.021
		P_{FD}	0.426	28.213	0
		P_{CWT-S3}	0.513	32.000	-0.446
		P_{CWT-S4}	0.331	18.000	0.396
		P_{CWT-S5}	0.272	14.636	0.592
	LNC_b	P_0	0.890	68.635	-3.362
		P_{REF}	0.913	70.527	-3.591
		$P_{PROCOSINE}$	0.934	72.251	-3.807
		P_{FD}	0.918	70.932	-3.641
		P_{CWT-S3}	0.873	67.238	-3.196
		P_{CWT-S4}	0.946	73.200	-3.928
		P_{CWT-S5}	0.383	22.589	0.191
LSTM	LNC_a	P_0	0.438	27.515	-0.054
		P_{REF}	0.518	30.442	-0.477
		$P_{PROCOSINE}$	0.435	27.536	-0.041
		P_{FD}	0.428	27.845	-0.009

		P_{CWT-S3}	0.427	28.077	-0.005
		P_{CWT-S4}	0.429	28.282	-0.012
		P_{CWT-S5}	0.431	27.733	-0.023
	LNC_b	P_0	0.930	71.906	-3.763
		P_{REF}	0.905	69.824	-3.505
		$P_{PROCOSINE}$	0.914	70.605	-3.601
		P_{FD}	0.918	70.923	-3.640
		P_{CWT-S3}	0.899	69.319	-3.444
		P_{CWT-S4}			
		P_{CWT-S5}	0.946	73.208	-3.929
LGBM	LNC_a	P_0	1.022	0.600	-4.748
		P_{REF}	0.432	0.238	-0.028
		$P_{PROCOSINE}$	0.499	0.285	-0.370
		P_{FD}	1.035	0.624	-4.902
		P_{CWT-S3}	0.879	0.518	-3.249
		P_{CWT-S4}	0.726	0.415	-1.899
		P_{CWT-S5}	0.731	0.412	-1.943
	LNC_b	P_0	0.435	0.304	-0.043
		P_{REF}	2.292	1.561	-27.923
		$P_{PROCOSINE}$	0.501	0.321	-0.379
		P_{FD}	1.251	0.809	-7.620
		P_{CWT-S3}	1.142	0.717	-6.173
		P_{CWT-S4}	1.150	0.727	-6.275
		P_{CWT-S5}	1.349	0.858	-9.012
SVR	LNC_a	P_0	0.324	0.171	0.424
		P_{REF}	0.315	0.201	0.453
		$P_{PROCOSINE}$	0.306	0.192	0.483
		P_{FD}	0.356	0.220	0.303
		P_{CWT-S3}	0.339	0.203	0.366
		P_{CWT-S4}	0.453	0.285	-0.130
		P_{CWT-S5}	0.620	0.371	-1.113
	LNC_b	P_0	5.258	3.779	-151.189
		P_{REF}	0.641	0.326	-1.262
		$P_{PROCOSINE}$	8.777	6.398	-423.080
		P_{FD}	0.488	0.258	-0.312

		P_{CWT-S3}	1.026	0.662	-4.797
		P_{CWT-S4}	1.200	0.804	-6.924
		P_{CWT-S5}	0.819	0.480	-2.694
Lasso	LNC_a	P_0	0.404	0.287	0.101
		P_{REF}	0.412	0.295	0.068
		$P_{PROCOSINE}$	0.432	0.313	-0.027
		P_{FD}	0.431	0.276	-0.024
		P_{CWT-S3}	0.467	0.345	-0.200
		P_{CWT-S4}	0.490	0.365	-0.323
		P_{CWT-S5}	0.391	0.284	0.158
	LNC_b	P_0	0.977	0.757	-4.257
		P_{REF}	0.977	0.757	-4.257
		$P_{PROCOSINE}$	0.977	0.757	-4.257
		P_{FD}	0.977	0.757	-4.257
		P_{CWT-S3}	0.977	0.757	-4.257
		P_{CWT-S4}	0.977	0.757	-4.257
		P_{CWT-S5}	0.714	0.548	-1.803

Table S2: The LNC estimation accuracy of non-parametric regression method based on T100 data.

Model	Evaluation indicator	Data preprocessing	RMSE	MAPE (%)	R^2
Conv-Transformer	LNC_a	P_0	0.363	20.605	0.273
		P_{REF}	0.336	19.147	0.377
		$P_{PROCOSINE}$	0.315	17.732	0.455
		P_{FD}	0.472	33.758	-0.224
		P_{CWT-S3}	0.297	16.918	0.515
		P_{CWT-S4}	0.247	13.811	0.665
		P_{CWT-S5}	0.353	19.760	0.315
	LNC_b	P_0	0.655	37.012	-1.363
		P_{REF}	0.802	45.181	-2.539
		$P_{PROCOSINE}$	1.084	61.782	-5.464
		P_{FD}	0.442	30.930	-0.076
		P_{CWT-S3}	0.385	23.211	0.184
		P_{CWT-S4}	0.547	33.238	-0.648
		P_{CWT-S5}	0.665	42.260	-1.436

ETransformer	LNC_a	P_0	0.552	31.556	-0.679
		P_{REF}	0.430	23.749	-0.016
		$P_{PROCOSINE}$	0.351	19.181	0.320
		P_{FD}	0.484	34.898	-0.291
		P_{CWT-S3}	0.440	25.631	-0.068
		P_{CWT-S4}	0.345	18.935	0.346
		P_{CWT-S5}	0.291	16.082	0.532
	LNC_b	P_0	0.654	49.228	-1.356
		P_{REF}	0.602	44.923	-0.998
		$P_{PROCOSINE}$	0.572	42.404	-0.802
		P_{FD}	0.442	30.880	-0.074
		P_{CWT-S3}	0.437	30.364	-0.052
		P_{CWT-S4}	0.443	30.981	-0.078
		P_{CWT-S5}	0.451	31.797	-0.117
LSTM	LNC_a	P_0	0.507	28.234	-0.417
		P_{REF}	0.444	27.380	-0.087
		$P_{PROCOSINE}$	0.432	27.595	-0.027
		P_{FD}	0.474	33.972	-0.237
		P_{CWT-S3}	0.456	32.355	-0.146
		P_{CWT-S4}	0.443	30.988	-0.079
		P_{CWT-S5}	0.428	27.799	-0.010
	LNC_b	P_0	0.623	46.645	-1.137
		P_{REF}	0.603	44.982	-1.002
		$P_{PROCOSINE}$	0.581	43.158	-0.860
		P_{FD}	0.447	31.454	-0.101
		P_{CWT-S3}	0.431	29.606	-0.024
		P_{CWT-S4}	0.449	31.644	-0.110
		P_{CWT-S5}	0.468	33.387	-0.204
LGBM	LNC_a	P_0	0.878	0.578	-4.372
		P_{REF}	0.396	0.215	0.135
		$P_{PROCOSINE}$	0.386	0.244	0.180
		P_{FD}	0.735	0.407	-1.974
		P_{CWT-S3}	0.439	0.226	-0.061
		P_{CWT-S4}	0.430	0.215	-0.018
		P_{CWT-S5}	0.531	0.301	-0.549

	LNC_b	P_0	0.381	0.209	0.201
		P_{REF}	1.735	1.142	-15.568
		$P_{PROCOSINE}$	0.387	0.235	0.177
		P_{FD}	1.012	0.609	-4.640
		P_{CWT-S3}	1.104	0.683	-5.712
		P_{CWT-S4}	1.117	0.695	-5.872
		P_{CWT-S5}	0.954	0.566	-4.009
SVR	LNC_a	P_0	0.356	0.201	0.302
		P_{REF}	0.286	0.157	0.550
		$P_{PROCOSINE}$	0.287	0.160	0.547
		P_{FD}	0.422	0.253	0.022
		P_{CWT-S3}	0.417	0.241	0.042
		P_{CWT-S4}	0.312	0.184	0.463
		P_{CWT-S5}	0.250	0.134	0.655
	LNC_b	P_0	2.229	1.378	-26.358
		P_{REF}	4.711	3.318	-121.168
		$P_{PROCOSINE}$	2.259	1.408	-27.083
		P_{FD}	0.402	0.203	0.109
		P_{CWT-S3}	1.003	0.682	-4.538
		P_{CWT-S4}	0.717	0.461	-1.832
		P_{CWT-S5}	1.070	0.702	-5.300
Lasso	LNC_a	P_0	0.364	0.230	0.270
		P_{REF}	0.355	0.234	0.305
		$P_{PROCOSINE}$	0.365	0.248	0.267
		P_{FD}	0.463	0.329	-0.178
		P_{CWT-S3}	0.435	0.318	-0.042
		P_{CWT-S4}	0.433	0.314	-0.032
		P_{CWT-S5}	0.293	0.195	0.529
	LNC_b	P_0	0.703	0.533	-1.723
		P_{REF}	0.646	0.486	-1.299
		$P_{PROCOSINE}$	0.631	0.473	-1.193
		P_{FD}	0.463	0.329	-0.178
		P_{CWT-S3}	0.456	0.323	-0.144
		P_{CWT-S4}	0.472	0.338	-0.224
		P_{CWT-S5}	0.480	0.353	-0.270

Table S3: The LNC estimation accuracy of parametric regression method based on all simulated data.				
Evaluation indicator	VI	RMSE	MAPE (%)	R^2
LNC_a	NDRE	0.539	0.376	-0.601
	ND705	0.464	0.339	-0.186
	TCARI	0.539	0.404	-0.599
	mND705	0.549	0.405	-0.658
	R705	0.496	0.365	-0.352
	R434	0.562	0.413	-0.741
	EVI	0.448	0.248	-0.103
	GARI	0.341	0.236	0.361
	GNDVI	0.341	0.237	0.36
	GRVI	0.308	0.201	0.477
	MCARI	0.559	0.419	-0.722
	MTVI1	0.435	0.308	-0.044
	NDVI	0.444	0.249	-0.086
	NDWI	0.43	0.276	-0.02
	PSRI	0.598	0.446	-0.965
	SR(800,680)	0.639	0.322	-1.249
	SR(750,705)	0.476	0.333	-0.245
	SR(708,775)	0.453	0.332	-0.129
	CI(800,550)	0.313	0.207	0.461
	CI(800,710)	0.522	0.353	-0.501
	mSR(800,680)	0.431	0.276	-0.024
	mSR(750,705)	0.511	0.334	-0.435
	mSR(708,775)	0.484	0.358	-0.292
	mCI(800,550)	0.426	0.273	0
	mCI(800,710)	0.609	0.398	-1.043
	SIPI	0.421	0.272	0.023
	Macc01	0.579	0.431	-0.846
	MTCI	0.949	0.598	-3.958
	DATT	0.575	0.427	-0.82
	VREI2	0.858	0.543	-3.055
LNC_b	NDRE	0.975	0.756	-4.237

ND705	0.976	0.756	-4.241
TCARI	0.976	0.756	-4.239
mND705	0.975	0.756	-4.237
R705	0.976	0.756	-4.242
R434	0.976	0.757	-4.248
EVI	0.978	0.758	-4.266
GARI	0.977	0.757	-4.251
GNDVI	0.977	0.757	-4.251
GRVI	0.977	0.757	-4.254
MCARI	0.977	0.757	-4.251
MTVI1	0.977	0.757	-4.25
NDVI	0.978	0.758	-4.265
NDWI	0.976	0.756	-4.248
PSRI	0.976	0.756	-4.239
SR(800,680)	0.979	0.759	-4.277
SR(750,705)	0.976	0.756	-4.239
SR(708,775)	0.976	0.756	-4.242
CI(800,550)	0.977	0.757	-4.253
CI(800,710)	0.975	0.756	-4.237
mSR(800,680)	0.977	0.757	-4.257
mSR(750,705)	0.971	0.752	-4.191
mSR(708,775)	0.974	0.755	-4.226
mCI(800,550)	0.977	0.757	-4.256
mCI(800,710)	0.972	0.753	-4.201
SIPI	0.977	0.757	-4.257
Macc01	0.975	0.756	-4.236
MTCI	0.974	0.754	-4.218
DATT	0.975	0.756	-4.236
VREI2	0.974	0.754	-4.223

Table S4: The LNC estimation accuracy of parametric regression method based on T100 data.

Evaluation indicator	VI	RMSE	MAPE (%)	R^2
LNC_a	NDRE	0.33	0.208	0.402
	ND705	0.31	0.202	0.472
	TCARI	0.352	0.241	0.319
	mND705	0.345	0.235	0.346
	R705	0.35	0.231	0.326
	R434	0.435	0.296	-0.042
	EVI	0.484	0.249	-0.288
	GARI	0.333	0.174	0.39
	GNDVI	0.321	0.169	0.432
	GRVI	0.354	0.182	0.311
	MCARI	0.424	0.306	0.011
	MTVI1	0.401	0.273	0.113
	NDVI	0.891	0.508	-3.371
	NDWI	0.47	0.281	-0.218
	PSRI	0.468	0.336	-0.207
	SR(800,680)	1.011	0.599	-4.632
	SR(750,705)	0.308	0.193	0.479
	SR(708,775)	0.303	0.199	0.494
	CI(800,550)	0.344	0.179	0.349
	CI(800,710)	0.317	0.196	0.446
	mSR(800,680)	0.453	0.274	-0.131
	mSR(750,705)	0.337	0.198	0.376
	mSR(708,775)	0.312	0.208	0.466
	mCI(800,550)	0.445	0.266	-0.091
	mCI(800,710)	0.358	0.215	0.293
	SIPI	0.735	0.387	-1.977
	Macc01	0.371	0.259	0.243
	MTCI	0.625	0.367	-1.148
	DATT	0.367	0.255	0.26
	VREI2	0.554	0.326	-0.692
LNC_b	NDRE	0.752	0.572	-2.113
	ND705	0.753	0.573	-2.119

TCARI	0.748	0.568	-2.076
mND705	0.75	0.57	-2.094
R705	0.751	0.572	-2.108
R434	0.749	0.57	-2.088
EVI	0.739	0.563	-2.006
GARI	0.759	0.578	-2.167
GNDVI	0.758	0.577	-2.164
GRVI	0.759	0.578	-2.175
MCARI	0.741	0.563	-2.022
MTVI1	0.734	0.557	-1.964
NDVI	0.747	0.569	-2.074
NDWI	0.758	0.578	-2.161
PSRI	0.747	0.568	-2.069
SR(800,680)	0.731	0.556	-1.94
SR(750,705)	0.754	0.573	-2.127
SR(708,775)	0.753	0.573	-2.118
CI(800,550)	0.759	0.578	-2.171
CI(800,710)	0.753	0.573	-2.123
mSR(800,680)	0.756	0.576	-2.144
mSR(750,705)	0.752	0.571	-2.109
mSR(708,775)	0.75	0.57	-2.097
mCI(800,550)	0.756	0.576	-2.147
mCI(800,710)	0.75	0.57	-2.099
SIPI	0.773	0.59	-2.285
Macc01	0.748	0.569	-2.082
MTCI	0.746	0.566	-2.06
DATT	0.749	0.569	-2.084
VREI2	0.748	0.568	-2.083

Table S5: The LNC estimation accuracy of parametric regression method based on all simulated data.

Evaluation indicator	VI	RMSE	MAPE (%)	R^2
LNC_a	mSR(800,680,1725)	0.431	0.276	-0.024
	mSR(750,705,1725)	0.511	0.334	-0.435
	mSR(708,775,1725)	0.484	0.358	-0.292
	mSR(800,680,445)	0.431	0.276	-0.024
	mSR(750,705,445)	0.606	0.396	-1.018
	mSR(708,775,445)	0.52	0.386	-0.489
LNC_b	mSR(800,680,1725)	0.977	0.757	-4.257
	mSR(750,705,1725)	0.971	0.752	-4.191
	mSR(708,775,1725)	0.974	0.755	-4.226
	mSR(800,680,445)	0.977	0.757	-4.257
	mSR(750,705,445)	0.975	0.755	-4.233
	mSR(708,775,445)	0.976	0.756	-4.239

Table S6. Gridsearchcv hyperparameter values

Algorithm	Hyperparameters
LGBM	n_estimators: [100, 300, 500], max_depth: [-1, 5, 10], learning_rate: [0.01, 0.05, 0.1], num_leaves: [31, 63, 127], min_child_samples: [20, 50], subsample: [0.8, 1.0], colsample_bytree: [0.8, 1], reg_alpha: [0, 0.1], reg_lambda: [0, 0.1], boosting_type: [gbdt]
SVR	C: [0.01, 0.1, 1], epsilon: [0.01, 0.1, 1], kernel: [poly, rbf, sigmoid], degree: [2, 3, 5], coef0: [0, 0.1, 0.5, 1], tol: [1e-4], max_iter: [500]
Lasso	alpha: [0.01, 0.1, 1], tol: [1e-3, 1e-4], max_iter: [500], selection: [cyclic, random]

Table S7. Summary statistics on maize leaf traits from simulated and in-situ data.

		LNC	LNC_a	LNC_b	C_w	C_m	C_{ab}
Simulated data n=200000	Mean	-	1.4292	2.3743	0.0225	0.0063	42.5012
	Min	-	0.1706	0.2257	0.0050	0.0006	5.0000
	Max	-	2.6821	4.5147	0.0400	0.0120	80.0000
	Std.	-	0.5380	1.2374	0.0101	0.0028	21.6479
in-situ data n=1724	Mean	1.4949	-	-	0.0042	0.0056	36.2589

	Min	0.3588	-	-	0.0010	0.0011	9.8189
	Max	2.6169	-	-	0.0072	0.0097	59.4776
	Std.	0.4263	-	-	0.0005	0.0007	10.1536

Table S8. Summary statistics on different crop leaf traits from in-situ data.

Crop		LNC	C_w	C_m	C_{ab}
Wheat n=231	Mean	1.4641	0.0152	0.0046	50.9493
	Min	0.5400	0.0089	0.0027	16.2670
	Max	2.5379	0.0203	0.0062	77.6670
	Std.	0.4216	0.0022	0.0005	9.5792
Rice n=252	Mean	1.4286	0.0105	0.0054	34.2564
	Min	0.4341	0.0074	0.0036	17.5696
	Max	2.1147	0.0136	0.0073	50.7691
	Std.	0.2759	0.0011	0.0007	6.2169
Sorghum n=646	Mean	1.5990	0.0178	0.0053	49.3054
	Min	0.4442	0.0067	0.0030	16.2105
	Max	2.6813	0.0390	0.0118	72.2620
	Std.	0.5769	0.0048	0.0015	14.5913

Table S9. The LNC estimation accuracy of the Transformer model trained on the T100 dataset for different crops.

**Crop**	**Scale**	**Evaluation indicator**	**The sample number of T100**	**Batch size**	**Model**	**RMSE**	**MAPE (%)**	**R^2**
Corn	16	LNC_a	11349	64	Conv-Transformer	0.247	13.811	0.665
					ETransformer	0.345	18.935	0.346

		LNC_b	8338	64	Conv-Transformer	0.547	33.238	-0.648
					ETransformer	0.443	30.981	-0.078
Wheat	16	LNC_a	5737	64	Conv-Transformer	0.209	11.920	0.754
					ETransformer	0.250	15.154	0.646
		LNC_b	3931	32	Conv-Transformer	0.426	25.159	-0.023
					ETransformer	0.425	25.204	-0.020
Rice	8	LNC_a	4254	16	Conv-Transformer	0.172	10.319	0.609
					ETransformer	0.200	11.720	0.475
		LNC_b	2158	16	Conv-Transformer	0.331	18.361	-0.444
					ETransformer	0.349	19.221	-0.611
Sorghum	32	LNC_a	4397	32	Conv-Transformer	0.412	19.657	0.488
					ETransformer	0.475	32.757	0.322
		LNC_b	6514	64	Conv-Transformer	0.845	50.579	-1.150
					ETransformer	0.813	34.931	-0.986

Table S10. The LNC estimation accuracy of the Transformer model trained on the entire dataset for different crops.

Crop	Scale	Evaluation indicator	Model	RMSE	MAPE (%)	R^2
Corn	16	LNC_a	Conv-Transformer	0.265	13.934	0.614
			ETransformer	0.331	18.000	0.396
		LNC_b	Conv-Transformer	0.261	14.145	0.625
			ETransformer	0.946	73.200	-3.928
Wheat	16	LNC_a	Conv-Transformer	0.346	18.770	0.323
			ETransformer	0.315	18.167	0.440
		LNC_b	Conv-Transformer	0.279	16.345	0.561
			ETransformer	0.868	66.384	-3.255

Rice	8	LNC_a	Conv-Transformer	0.195	11.458	0.499
			ETransformer	0.390	25.135	-1.009
		LNC_b	Conv-Transformer	0.920	68.611	-10.167
			ETransformer	0.840	62.480	-8.309
Sorghum	32	LNC_a	Conv-Transformer	0.424	17.274	0.460
			ETransformer	0.491	32.601	0.275
		LNC_b	Conv-Transformer	0.600	32.002	-0.082
			ETransformer	0.929	73.700	-1.595

Table S11. The LNC estimation accuracy of the PROCWT model for different crops.

Crop	Scale	Evaluation indicator	RMSE	MAPE (%)	R^2
Corn	16	LNC_a	0.290	17.227	0.536
		LNC_b	0.517	27.960	-0.472
Wheat	16	LNC_a	0.235	12.992	0.687
		LNC_b	0.541	30.863	-0.652
Rice	8	LNC_a	0.176	10.553	0.591
		LNC_b	0.627	40.368	-4.182
Sorghum	32	LNC_a	0.511	34.898	0.215
		LNC_b	0.862	39.542	-1.237

Table S12. VIs used for LNC estimation.

Index	Formula	Reference
NDRE	$\frac{R_{790} - R_{720}}{R_{790} + R_{720}}$	[1]
ND705	$\frac{R_{750} - R_{705}}{R_{750} + R_{705}}$	[2]

TCARI	$3[(R_{700}-R_{670})-0.2(R_{700}-R_{550})(\frac{R_{700}}{R_{670}})]$	[3]
mND705	$\frac{R_{750}-R_{705}}{R_{750}+R_{705}-2R_{445}}$	[2]
Modified Simple Difference 705 Index (R705)	$\frac{R_{705}}{R_{717}+R_{491}}$	[4]
Modified Simple Difference 434 Index (R434)	$\frac{R_{434}}{R_{496}+R_{401}}$	[4]
EVI	$2.5\times\frac{R_{872}-R_{661}}{R_{872}+6\times R_{661}-7.5\times R_{488}+1}$	[5]
GARI	$\frac{R_{872}-[R_{559}-(R_{488}-R_{661})]}{R_{872}+[R_{559}-(R_{488}-R_{661})]}$	[6]
GNDVI	$(R_{872}-R_{559})/(R_{872}+R_{559})$	[7]
GRVI	R_{872}/R_{559}	[8]
MCARI	$\frac{[(R_{702}-R_{671})-0.2\times(R_{702}-R_{549})]\times R_{702}}{R_{671}}$	[9]
MTVI1	$1.2\times[1.2\times(R_{800}-R_{550})-2.5\times(R_{670}-R_{550})]$	[10]
NDVI	$(R_{800}-R_{670})/(R_{800}+R_{670})$	[11]
NDWI	$(R_{872}-R_{1245})/(R_{872}+R_{1245})$	[12]
PSRI	$(R_{680}-R_{500})/R_{750}$	[13]
$SR_{800,680}$	R_{800}/R_{680}	[2]
$SR_{750,705}$	R_{750}/R_{705}	[2]
$SR_{708,775}$	R_{708}/R_{775}	[14]
$CI_{800,550}$	$R_{800}/R_{550}-1$	[15]
$CI_{800,710}$	$R_{800}/R_{710}-1$	[15]
$mSR_{800,680}$	$(R_{800}-R_{1925})/(R_{680}-R_{1925})$	This study
$mSR_{750,705}$	$(R_{750}-R_{1925})/(R_{705}-R_{1925})$	This study
$mSR_{708,775}$	$(R_{708}-R_{1925})/(R_{775}-R_{1925})$	This study

$mCI_{800,550}$	$(R_{800}-R_{1925})/(R_{550}-R_{1925})-1$	This study
$mCI_{800,710}$	$(R_{800}-R_{1925})/(R_{710}-R_{1925})-1$	This study
SIPI	$(R_{800}-R_{445})/(R_{800}-R_{680})$	[16]
Macc01	$(R_{780}-R_{710})/(R_{780}-R_{680})$	[17]
MTCI	$(R_{754}-R_{709})/(R_{709}-R_{681})$	[18]
DATT	$(R_{850}-R_{710})/(R_{850}-R_{680})$	[19]
VREI2	$(R_{734}-R_{747})/(R_{715}-R_{726})$	[20]

Note: CI=Chl indice.

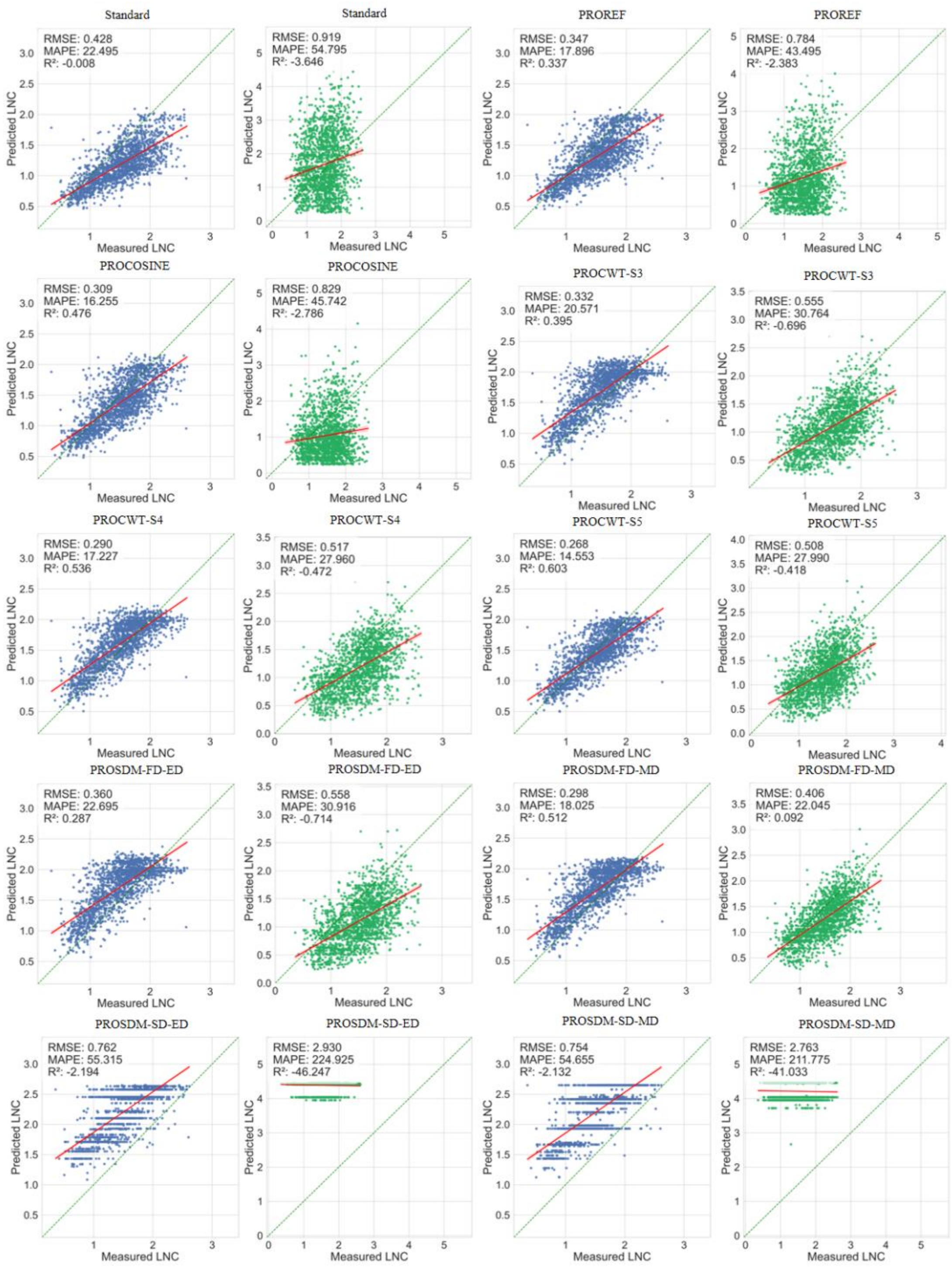

Fig. S1. LNC estimation accuracy based on physical models. Blue points represent the LNC of simulated spectra based on the nitrogen allocation model, while green points represent the LNC of simulated spectra based on the protein-to-nitrogen conversion model. The green dashed line represents the 1:1 line, and the red solid line represents the fitted line.

REFERENCES

[1] Rodriguez, D., G. Fitzgerald, R. Belford, and L. Christensen, *Detection of nitrogen deficiency in wheat from spectral reflectance indices and basic crop eco-physiological concepts.* Australian Journal of Agricultural Research, 2006. **57**(7): p. 781-789.

[2] Sims, D.A. and J.A. Gamon, *Relationships between leaf pigment content and spectral reflectance across a wide range of species, leaf structures and developmental stages.* Remote sensing of environment, 2002. **81**(2-3): p. 337-354.

[3] Haboudane, D., J.R. Miller, N. Tremblay, P.J. Zarco-Tejada, and L. Dextraze, *Integrated narrow-band vegetation indices for prediction of crop chlorophyll content for application to precision agriculture.* Remote sensing of environment, 2002. **81**(2-3): p. 416-426.

[4] Tian, Y., X. Yao, J. Yang, W. Cao, D. Hannaway, and Y. Zhu, *Assessing newly developed and published vegetation indices for estimating rice leaf nitrogen concentration with ground-and space-based hyperspectral reflectance.* Field Crops Research, 2011. **120**(2): p. 299-310.

[5] Liu, H.Q. and A. Huete, *A feedback based modification of the NDVI to minimize canopy background and atmospheric noise.* IEEE transactions on geoscience and remote sensing, 1995. **33**(2): p. 457-465.

[6] Gitelson, A.A., Y.J. Kaufman, and M.N. Merzlyak, *Use of a green channel in remote sensing of global vegetation from EOS-MODIS.* Remote sensing of Environment, 1996. **58**(3): p. 289-298.

[7] Ma, B., M.J. Morrison, and L.M. Dwyer, *Canopy light reflectance and field greenness to assess nitrogen fertilization and yield of maize.* Agronomy Journal, 1996. **88**(6): p. 915-920.

[8] Tucker, C.J., *Red and photographic infrared linear combinations for monitoring vegetation.* Remote sensing of Environment, 1979. **8**(2): p. 127-150.

[9] Wu, C., Z. Niu, Q. Tang, and W. Huang, *Estimating chlorophyll content from hyperspectral vegetation indices: Modeling and validation.* Agricultural and forest meteorology, 2008. **148**(8-9): p. 1230-1241.

[10] Haboudane, D., J.R. Miller, E. Pattey, P.J. Zarco-Tejada, and I.B. Strachan, *Hyperspectral vegetation indices and novel algorithms for predicting green LAI of crop canopies: Modeling and validation in the context of precision agriculture.* Remote sensing of environment, 2004. **90**(3): p. 337-352.

[11] Rouse, J.W., R.H. Haas, J.A. Schell, and D.W. Deering, *Monitoring vegetation systems in the Great Plains with ERTS.* NASA Spec. Publ, 1974. **351**(1): p. 309.

[12] Gao, B.-C., *NDWI—A normalized difference water index for remote sensing of vegetation liquid water from space.* Remote sensing of environment, 1996. **58**(3): p. 257-266.

[13] Merzlyak, M.N., A.A. Gitelson, O.B. Chivkunova, and V.Y. Rakitin, *Non-destructive optical detection of pigment changes during leaf senescence and fruit ripening.* Physiologia plantarum, 1999. **106**(1): p. 135-141.

[14] Féret, J.-B., C. François, A. Gitelson, G.P. Asner, K.M. Barry, C. Panigada, A.D. Richardson, and S. Jacquemoud, *Optimizing spectral indices and chemometric analysis of leaf chemical properties using radiative transfer modeling.* Remote sensing of environment, 2011. **115**(10): p. 2742-2750.

[15] Gitelson, A.A., A. Viña, V. Ciganda, D.C. Rundquist, and T.J. Arkebauer, *Remote estimation of canopy chlorophyll content in crops.* Geophysical research letters, 2005. **32**(8).

[16] Penuelas, J., F. Baret, and I. Filella, *Semi-empirical indices to assess carotenoids/chlorophyll a ratio from leaf spectral reflectance.* Photosynthetica, 1995. **31**(2): p. 221-230.

[17] Maccioni, A., G. Agati, and P. Mazzinghi, *New vegetation indices for remote measurement of chlorophylls based on leaf directional reflectance spectra.* Journal of Photochemistry and Photobiology B: Biology, 2001. **61**(1-2): p. 52-61.

[18] Dash, J. and P. Curran, *The MERIS terrestrial chlorophyll index.* 2004.

[19] Datt, B., *A new reflectance index for remote sensing of chlorophyll content in higher plants: tests using Eucalyptus leaves.* Journal of plant physiology, 1999. **154**(1): p. 30-36.

[20] Vogelmann, J., B. Rock, and D. Moss, *Red edge spectral measurements from sugar maple leaves.* TitleREMOTE SENSING, 1993. **14**(8): p. 1563-1575.
